# Tryptophan–kynurenine metabolic reprogramming along the gut–brain axis alleviates Alzheimer’s pathology

**DOI:** 10.1186/s12974-026-03796-1

**Published:** 2026-04-24

**Authors:** Hyunjung Choi, Seok Beom Hong, Yumi Kim, Hyunchae Joung, Yukyung Choi, Jiah Cha, Ji Yong Park, Yun-Sang Lee, Hayoung Choi, Jong Won Han, Kyung Hwan Kim, Chang Hun Shin, Do Yup Lee, Inhee Mook-Jung

**Affiliations:** 1 Convergence Dementia Research Center, Medical Research Center, Seoul National University, Seoul, 03080 Republic of Korea; 2 Department of Biomedical Science, College of Medicine, Seoul National University, Seoul, 03080 Republic of Korea; 3 Department of Agricultural Biotechnology, Seoul National University, Seoul, Republic of Korea; 4 Central Research Institute, Chong Kun Dang Bio, Ansan, 15604 Republic of Korea; 5 Department of Nuclear Medicine, Seoul National University Hospital, Seoul, 03080 Republic of Korea; 6 Cancer Research Institute, Seoul National University, Seoul, 03080 Republic of Korea; 7 Department of Nuclear Medicine, College of Medicine, Seoul National University, Seoul, 03080 Republic of Korea; 8 Institute of Radiation Medicine, Medical Research Center, College of Medicine, Seoul National University, Seoul, 03080 Republic of Korea; 9 Department of Molecular Medicine and Biopharmaceutical Sciences, Graduate School of Convergence Science and Technology, Seoul National University, 08826 Seoul, Republic of Korea; 10 Center for Food and Bioconvergence, Research Institute for Agricultural and Life Sciences, Interdisciplinary Programs in Agricultural Genomics, Seoul National University, Seoul, 08826 Republic of Korea

**Keywords:** Alzheimer’s disease, Gut microbiota, Probiotics, Kynurenic acid, Neuroprotection, Neuroinflammation, Gut–brain axis, Multi-omics

## Abstract

The gut–brain axis influences neuroinflammation and metabolic homeostasis in Alzheimer’s disease (AD). Disruption of gut microbiota and barrier function promotes amyloid and tau pathology via immune and metabolic dysregulation. In this study, *Limosilactobacillus fermentum* SRK414 (SRK414) was orally administered to ADLP^APT^ mice, resulting in reduced Aβ and tau pathology and improved cognition. Multi-omics analysis revealed that SRK414 altered gut microbial composition and increased hippocampal kynurenic acid (KYNA), a metabolite linked to neuroimmune regulation. Increased hippocampal KYNA was associated with metabolic changes consistent with enhanced neuronal fatty acid oxidation, reduced lipid accumulation, and suppressed microglial activation, suggesting improved hippocampal homeostasis. In vitro studies further showed that KYNA attenuated tau-related and inflammatory phenotypes. These findings support a link between gut microbial modulation and brain resilience, and suggest that KYNA may contribute to the neuroprotective effects associated with SRK414 treatment. This study highlights metabolites modulated by SRK414 administration as potential mediators of microbiota-based therapeutic effects in AD.

**Supplementary Information:**

The online version contains supplementary material available at 10.1186/s12974-026-03796-1.

## Introduction

The gut microbiota has emerged as a critical determinant of host health and disease, with growing evidence linking it to brain function and mental health [1, 2]. These effects are mediated through microbial metabolites [3], immune modulation [4], vagus nerve stimulation [5], and neurotransmitter regulation [6]. Disruption of this network through altered gut microbiota composition has been associated with behavioral abnormalities and neuropsychiatric symptoms [7–9]. Alterations in gut microbiota composition have also been associated with various neurological disorders, including schizophrenia, autism spectrum disorder [10]. Moreover, it also impairs intestinal barrier function, enabling microbial translocation and systemic immune activation, which triggers a chronic neuroinflammatory cascade [11]. These processes are increasingly implicated in the pathogenesis of neurodegenerative disease by exacerbating pathological protein aggregation [11, 12].

In Alzheimer’s disease (AD), altered gut microbiota composition and increased intestinal permeability have been reported to facilitate the translocation of pro-inflammatory molecules into the circulation, potentially contributing to systemic inflammation, which has been associated with amyloid and tau pathology. In addition, altered microbial metabolite profiles have been closely associated with disease progression [13–16]. Germ-free and antibiotic-treated mouse models show reduced amyloid-beta deposition [17, 18]. Transplantation of microbiota from healthy individuals, AD patients or disease models can modulate AD pathology [13, 19], indicating a causal role for gut microbes through metabolite production and host signaling pathways. These findings collectively highlight the gut microbiota and intestinal barrier as modifiable therapeutic targets for attenuating AD pathology.

Probiotic supplementation offers a promising approach for restoring microbial balance and regulating immune and metabolic pathways across the gut-brain axis [20, 21]. However, their effects on both gut and brain compartments remain poorly characterized in AD models, particularly through integrative multi-omics approaches.

We investigated the therapeutic effects of *Limosilactobacillus fermentum* (*L. fermentum*) SRK414, a probiotic with antioxidant, anti-inflammatory, and gut barrier-enhancing properties [22], in the ADLP^APT^ mouse model of amyloid and tau pathologies [23] (hereafter referred to as SRK414). We assessed its impact on behavior, histology, and multi-omics profiles of gut and brain tissues to investigate the systemic and neuroprotective effects of SRK414.

Our results show that SRK414 reshapes gut microbiota, suppresses inflammation, restores metabolic and transcriptional homeostasis, and preserves gut barrier function, ultimately alleviating AD-related neuropathology and cognitive deficits. This study provides mechanistic insights into probiotic-mediated neuroprotection via the gut–brain axis and supports the therapeutic potential of microbiota-targeted interventions in AD.

## Materials and methods

### Animals

To generate an Alzheimer's disease mouse model exhibiting both amyloid-β and tau pathologies, female ADLP^APT^ transgenic mice were generated by crossing 5 × FAD mice (Tg6799; Jackson Laboratory, Stock #006554), expressing human APP with Swedish, Florida, and London mutations and human PSEN1 (M146L and L286V) under the Thy1 promoter, with JNPL3 mice (TauP301L-JNPL3; Taconic, Stock #2508), which express human tau harboring the P301L mutation under the prion protein promoter. Only female mice were used in this study, based on prior reports indicating that ADLP^APT^ females develop cognitive deficits and AD-like pathology earlier than males.

Experimental animals were divided into three groups: ADLP^WT^, ADLP^APT^ controls receiving vehicle treatment (PBS), and ADLP^APT^ mice treated with *Limosilactobacillus fermentum* (*L. fermentum*) SRK414. *L. fermentum* SRK414-treated group received oral gavage of 5.0 × 10⁹ CFU/mouse/day of *L. fermentum* SRK414 suspended in sterile phosphate-buffered saline (PBS), 5 days per week, starting at 10 weeks of age and continuing until 30 weeks of age. The control groups, including ADLP^WT^ and ADLP^APT^ mice, received an equivalent volume of PBS as vehicle. All mice were maintained on a standard chow diet and housed in separate sterile cages per group to eliminate cage effect-induced microbial homogenization.

All experimental procedures were conducted in accordance with the NIH Guide for the Care and Use of Laboratory Animals (NIH publication No. 85–23, revised 1985) and were approved by the Institutional Animal Care and Use Committee (IACUC) of Seoul National University.

### Probiotics preparation

Probiotics preparation was performed as previously described [24], with minor modifications.

*L. fermentum* SRK414 was originally isolated from infant feces. The strain was cultured in de Man, Rogosa, and Sharpe (MRS) broth (BD/Difco, Franklin Lakes, NJ, USA) under aerobic condition at 37℃ for 24 h. For long-term storage, the cultured broth was mixed with an equal volume of 20% glycerol solution and stored at − 80℃.

For seed preparation, *L. fermentum* SRK414 was pre-cultured in MRS broth at 37℃ for 24 h and subsequently transferred to an optimized medium in a mini jar fermenter (Bio Control & Science, MARADO-05D-PS, Xiamen, Fujian, China). Fermentation was carried out at 37℃ for 16–18 h with constant agitation at 120 rpm, while maintaining the pH at 5.5–6.0 using automatic titration with 25% (w/v) NaOH solution. After fermentation, cells were harvested by centrifugation at 6000 rpm for 10 min (Hanil, Supra R12, Daejeon, Republic of Korea), and the resulting concentrate (40 ×) was lyophilized using a freeze-dryer (Cooling & Heating System, Lab-Mast 10, Seoul, Republic of Korea) following the manufacturer’s protocol. The cell counts of the final probiotic powder were analyzed, and the powder was suspended in phosphate-buffered saline (PBS; Bioneer, Daejeon, Republic of Korea) adjusting to concentration of 5.0 × 10⁹ CFU per mouse for oral administration for animal experiment.

### Aβ ELISA analysis

To quantify human Aβ levels in the cortex, tissues from transgenic mice were perfused with PBS, homogenized on ice in RIPA buffer (50 mM Tris–HCl pH 7.4, 150 mM NaCl, 1% Nonidet P-40, 0.1% SDS, 0.5% deoxycholic acid, and protease inhibitor cocktail [P3840, Sigma-Aldrich]), and sonicated. The homogenates were centrifuged at 17,949 × g for 15 min at 4 °C, and the supernatants were used for protein quantification via BCA assay.

A total of 100 μg of protein was subjected to ultracentrifugation at 100,000 × g for 1 h at 4 °C to separate RIPA-soluble and -insoluble fractions. The RIPA-insoluble pellet was washed with PBS, centrifuged again under the same conditions, and subsequently resuspended in 70% formic acid. The sample was neutralized at a 1:20 ratio using 1 M Tris base (pH 11) prior to analysis. Human Aβ40 and Aβ42 concentrations in both fractions were measured using Aβ-specific ELISA kits according to the manufacturer’s instructions (27713, 27711; IBL).

### Immunohistochemistry

Mice were deeply anesthetized with Zoletil (Virbac, Carros, France) and transcardially perfused with phosphate-buffered saline (PBS). Brains were post-fixed in 4% paraformaldehyde (PFA) at 4 °C for 24 h, then cryoprotected in 30% sucrose solution for 3 days. Coronal brain Sects. (30 μm thickness) were prepared using a cryostat (Leica) and stored in cryoprotectant buffer.

Prior to immunostaining, brain sections were washed with PBS to remove residual storage buffer. To enable immunodetection of both amyloid-β and tau, antigen retrieval was carried out by incubating sections in 70% formic acid for 20 min. After PBS washing, sections were incubated for 1 h in blocking/permeabilization solution containing 0.3% Triton X-100, 0.5 mg/mL bovine serum albumin (BSA), and 5% normal horse serum (Vector Laboratories, S-2000–20) in PBS. The same buffer was used to prepare the primary antibody solution, and sections were incubated overnight at 4 °C with primary antibodies. Details of the primary antibodies are provided in Supplementary Table 1.

On the following day, sections were washed with PBS and then incubated with biotinylation reagents to label the primary antibodies. After thorough washing with PBS, biotinylated sections were incubated with appropriate secondary antibodies for 1 h at room temperature in PBS containing 5% normal serum. After additional PBS washes, nuclei were counterstained with DAPI (1 μg/mL) for 15 min. Finally, sections were mounted on glass slides using mounting medium and imaged using confocal microscopy (LSM700, Carl Zeiss; and ANDOR BC43, Oxford Instruments).

### Spontaneous alternation in Y-maze

Spatial working memory was assessed using the spontaneous alternation Y-maze test as previously described [25, 26]. Mice were placed at the end of one arm of a Y-shaped maze (each arm 30 cm in length) and allowed to explore freely for 8 min. An arm entry was scored when all four paws entered an arm. Spontaneous alternation was defined as consecutive entries into all three arms (e.g., ABC, BCA), and the percentage of spontaneous alternation was calculated as the ratio of actual to possible alternations. To ensure adequate exploration for reliable alternation estimation, mice with < 10 total arm entries were excluded from analysis.

### Novel object recognition test

Long-term object recognition memory was assessed by NOR test as previously described [27].

The NOR test was conducted over three consecutive days in a square open-field arena (40 × 40 cm) to assess recognition memory. On Day 1, mice were habituated to the empty arena for 30 min. On Day 2 (familiarization phase), two identical objects were placed in opposite corners of the arena, and mice were allowed to explore for 10 min. On Day 3 (test phase), one of the familiar objects was replaced with a novel object of similar size but different shape and texture, and mice were allowed to explore for 5 min. Exploratory behavior was recorded and recognition memory was quantified using the discrimination index, defined as the time spent exploring the novel object divided by the total time spent exploring both objects.

### 16S rRNA gene amplicon sequencing

16S rRNA gene amplicon sequencing was performed as previously described [28], with minor modifications.

The cecum samples were frozen at −80 °C before DNA extraction, and DNA was extracted using a DNeasy PowerSoil Kit (Qiagen, Hilden, Germany) according to the manufacturer’s instruction. Concentration of DNA was quantified using a Quanti-IT PicoGreen (Invitrogen, USA), and the Libraries were prepared using the Illumina 16S rRNA gene amplicon sequencing protocol (Illumina, San Diego, CA, USA). The V3 and V4 region of 16S rDNA gene were amplified using a universal primer pair with Illumina adapter overhang sequences (V3-F: 5’-TCGTCGGCAGCGTCAGATGTGTATAAGAGACAGCCTACGGGNGGCWGCAG-3’, V4-R: 5’-GTCTCGTGGGCTCGGAGATGTGTATAAGAGACAGGACTACHVGGGTATCTAATCC-3’) for the first amplifications, and the PCR product was purified with AMPure beads (Agencourt Bioscience, MA, USA). The final purified PCR product was then quantified using qPCR, and qualified using the TapeStation D1000 ScreenTape (Agilent Technologies, Waldbronn, Germany). The paired-end sequencing was performed using MiSeq system (Illumina, San Diego, USA) at Macrogen Inc. (Korea).

Taxonomic profiling of the OTUs was performed by assigning taxonomic labels using the SILVA reference databases [29], and sequence alignment was performed with the NCBI 16S BLAST database. The relative abundance of taxonomy was compared among the groups, and community diversity was analyzed using Shannon’s diversity index as α-diversity and Bray–Curtis diversity index as β-diversity by principal coordinate analysis (PCoA). Linear discriminant analysis Effect Size (LEfSe) analysis was performed using a Qiime2 platform [30].

### In vivo gut permeability imaging assay

In vivo intestinal permeability was assessed using RI-click-FITC-dextran, a radioisotope-labeled dextran-based tracer designed to overcome the limitations of conventional fluorescence-based quantification. To minimize interference from food intake, mice were fasted for 4 h prior to tracer administration. A single oral gavage of 50–100 µCi/mouse of RI-click-FITC-dextran was performed. This tracer incorporates a radioisotope (e.g., Cu-64 or Ga-68) into the dextran backbone via click chemistry, allowing for sensitive and quantitative detection with reduced variability from tissue autofluorescence or hair interference. Whole-body imaging was conducted using an in vivo imaging system (IVIS) at 10, 60 and 120 min post-administration to evaluate intestinal permeability.

### In vivo gut permeability serum FITC-dextran quantification

Gut permeability was assessed using a FITC–dextran assay as previously described [13, 31], with minor modifications. Mice were fasted for 4 h with food and water removed. FITC–dextran (4 kDa; FD4, Sigma-Aldrich) was administered orally at 0.6 mg/g. Two hours after administration, blood was collected via retro-orbital puncture and protected from light. Samples were incubated at room temperature for 30 min and centrifuged at 17,949 × g for 15 min at 4 °C. The supernatant (serum) was transferred to a new tube. A sample was loaded into a black, flat-bottom 96-well plate (Corning), and fluorescence was measured using a microplate reader (Excitation 490 nm, Emission 520 nm). All procedures were performed with minimal light exposure.

#### Generation of apical-out colon organoids from hiPSCs and FITC-based permeability assay

Human induced pluripotent stem cell (hiPSC)-derived colon organoids were generated as previously described [32], with minor modifications. In brief, hiPSCs were stepwise differentiated into definitive endoderm, hindgut, and colonic spheroids, followed by Matrigel-embedded three-dimensional culture and maturation to establish human colon organoids. After stabilization, mature organoids were released from Matrigel using cell recovery solution (354253, Corning) at 4 °C, washed with DPBS, and maintained in suspension culture in ultra-low attachment 96-well plates (7007, Corning). In the absence of extracellular matrix support, organoids were cultured for 4–7 days to allow inversion of epithelial polarity, resulting in apical surfaces facing outward. To induce epithelial barrier disruption, apical-out organoids were exposed to a cytokine mixture of TNF-α (AF-300–01, Peprotech) and IFN-γ (AF-300–02, Peprotech) (30 ng/mL each) for 48 h. After cytokine washout, organoids were treated with SRK414 for 24 h, followed by incubation with 1 mg/mL 4-kDa FITC-dextran (FD4, Sigma) for 1 h at 37 °C to assess epithelial barrier integrity. Organoids were then directly imaged without washing using confocal microscopy (BC43, Andor), and FITC-dextran fluorescence within the organoid area was quantified as an indicator of epithelial permeability.

#### Transcriptomic analysis

The RNA was extracted from the hippocampus and cortex of brain and colon samples, which were frozen at −80 °C prior to preparation, using a Qiagen RNeasy Kit (Qiagen). For the mRNA sequencing, the NovaSeq 6000 platform (Illumina) was employed to analyze the transcriptome. The library was constructed using the Truseq Stranded mRNA Library Prep Kit (Illumina), which allows for the generation of stranded RNA-Seq libraries, preserving the directional information of transcripts. Sequencing was performed utilizing paired-end read length of 100 base pairs, yielding high-quality reads that facilitate effective transcript reconstruction and quantification. After sequencing, a de novo assembly of the transcriptome was conducted using the Trinity program [33] allowing comprehensive transcript identification. Subsequently, differential expression analysis of genes (DEGs) analysis was performed using DESeq2 package [34], with a false discovery rate (FDR) threshold at 0.05. Gene set enrichment analysis (GSEA) was conducted with default parameters, using the Hallmark gene set collection from MSigDB used for pathway analysis using GSEA software [35].

#### Metabolite extraction

Brain samples were weighed and lyophilized until complete dryness. Lyophilized brain tissues were finely ground using a Mixer Mill MM400 (Retsch GmbH & Co., Germany) with a single steel bead (frequency 28 Hz, time 90 s). The powder samples were extracted with 1400 μL of cold extraction solvent (methanol: isopropanol: water, 3:3:2, v/v/v) and homogenized in Mixer Mill MM400 at frequency of 25 Hz for 90 s, followed by sonication (10 min) in ice and centrifugation (10 min, 13,200 × *g* at 4 °C). For short-chain fatty acids (SCFAs) analysis, 40 μL of the supernatant was aliquoted into separate 2-mL tubes. For untargeted metabolomics analysis, the 1300 μL of supernatant was transferred into new 1.5-mL tubes and concentrated to dryness using a speed vacuum concentrator (SCANVAC, Korea).

100 μl of serum samples and 100 mg of feces and cecal samples were extracted with 1400 μL of cold extraction solvent (methanol: isopropanol: water, 3:3:2, v/v/v) and homogenized in Mixer Mill MM400 at frequency of 25 Hz for 90 s, followed by sonication (10 min) and centrifugation (10 min, 13,200 × *g* at 4 °C). For short-chain fatty acids (SCFAs) analysis, 40 μL of the supernatant was aliquoted into separate 2-mL tubes. For untargeted metabolomics analysis, the 600 μl supernatant was transferred into new 1.5-mL tubes and concentrated to dryness using a speed vacuum concentrator (SCANVAC, Korea).\

Feeding material was extracted with 1400 μL of cold extraction solvent (methanol: isopropanol: water, 3:3:2, v/v/v) and homogenized in Mixer Mill MM400 at frequency of 25 Hz for 90 s, followed by sonication (10 min) and centrifugation (10 min, 13,200 × *g* at 4 °C). For untargeted metabolomics analysis, the 1300 μl supernatant was transferred into new 1.5-mL tubes and concentrated to dryness using a speed vacuum concentrator (SCANVAC, Korea).

#### Short Chain Fatty Acids (SCFA) analysis

Short Chain Fatty Acids Analysis was performed as previously described [36], with minor modifications.

The supernatant (40 μl) was mixed with 20 μl of 200 mM 3-nitrophenylhydrazine in 70% acetonitrile and 20 μl of a 1-Ethyl-3-(3-dimethylaminopropyl)carbodiimide (120 mM) dissolved in 70% acetonitrile with 6% pyridine. The mixture was incubated for 30 min at 40 °C. Fecal and cecal samples were diluted with 1.92 ml of 70% of acetonitrile. The derivatives were filtered through 0.2 μm PTFE filter (Analytical Service). Standards of SCFAs (acetate, propionate, butyrate, isobutyrate, 2-methylbutyrate, valerate, and isovalerate) were derivatized as described above.

The derivatives were chromatographically separated with a 150 × 2.1 mm UPLC BEH 1.7‐μm C18 column (Waters, Milford, MA, USA) equipped with 5.0 mm × 2.1 mm UPLC BEH 1.7 μm C18 VanGuard Pre‐Column (Waters) controlled by Vanquish UHPLC system (Thermo Fisher Scientific, Waltham, MA, USA). The mobile phase is composed of 0.1% formic acid in water (solvent A, v/v) and 0.1% formic acid in acetonitrile (solvent B, v/v) with a flow rate of 0.35 ml/min. The gradient of LC elution (0–15 min) was programmed as follows: 15% B for 2 min, 15–55% B gradient over 11 min, 100% B held for 2 min and re‐equilibration in 15% B for 3 min. Injection volume was 2 μl. Mass spectra were acquired using Q‐Exactive Focus Orbitrap (Thermo Fisher Scientific, Waltham) equipped with an electrospray ionization (ESI) interface (HESI‐II) in negative ionization mode, and the system was controlled using Xcalibur 4.0 and Q‐Exactive Tune software. Raw data were processed by Tracefinder software (version 4.1, Thermo Fisher Scientific, San José, CA, USA). A mass tolerance for precursor ion and retention time tolerance were set to 5 ppm and 0.5 min, respectively.

#### Untargeted metabolomics analysis

Untargeted Metabolomics Analysis was performed as previously described [36], with minor modifications.

The dried extracts were reconstituted with 70% acetonitrile for LC‐Orbitrap MS analysis. The samples were filtered through 0.2 μm PTFE filter (Analytical Service). Chromatographic separation was carried out using Ultmate‐3000 UPLC system (Thermo Fisher Scientific, Waltham) and a 100 × 2.1‐mm UPLC BEH 1.7‐μm C18 column (Waters) equipped with 5.0 mm × 2.1‐mm UPLC BEH 1.7 μm C18 VanGuard Pre‐Column (Waters). The mobile phase is composed of 0.1% formic acid in water (solvent A, v/v) and 0.1% formic acid in acetonitrile (solvent B, v/v) with a flow rate of 0.3 ml/min. The gradient of LC elution was programmed as follows: 0.5% for 0.1 min, 0.5%–80% gradient over 10 min, 99.5% held for 2 min and re‐equilibration in 0.5% for 3 min. All MS spectra were acquired on a Q-Exactive Plus-Orbitrap MS with heater temperature at 320 °C, sheath gas flow rate of 40 L/h, auxiliary gas flow rate of 10 L/h, sweep gas flow rate of 0 L/h, the capillary temperature at 300 °C, full scan range from 80 to 1,200, and data-dependent MS/MS scan (dd-MS^2^, Top *n* = 5). The full scan and MS/MS scan were collected at resolutions of 70,000 and 17,500 respectively. For low-abundance analyte, parallel reaction monitoring (PRM) was used for targeted MS/MS conformation by comparing their fragment ion spectra with the reference standard. Data acquisition and pre-processing were performed using Xcalibur software (Thermo Fisher Scientific, San José).

#### Data processing

The RAW data files were converted to.mzXML format using the ProteoWizard tool MSConvert and processed in MZmine 4.2.0 for feature list generation. The processing steps included centroid mass detection at MS1 and MS2 levels, with noise factors set to 5 and 2.5, respectively. Chromatogram building was performed with a minimum intensity of 1.0E4 and a scan-to-scan m/z tolerance of 5 ppm. Chromatogram smoothing was applied using the Savitzky-Golay algorithm with a retention time smoothing value of 5. The local minimum feature resolver was applied with a chromatographic threshold of 80% and a minimum peak top/edge ratio of 1.8. A 13 C isotope filter removed isotopic peaks with a 3 ppm m/z tolerance and a 0.04 min retention time tolerance, retaining only the monoisotopic peak. Peak alignment was conducted with an m/z tolerance of 5 ppm and a retention time tolerance of 0.5 min, followed by a duplicate peak filter set to 1.5 ppm and 0.07 min. Correlation grouping was performed with a retention time tolerance of 0.06. Spectral library searches were performed against public libraries (MS-DIAL-VS19, GNPS, MoNA experimental spectra) and in-house library, using a minimum cosine similarity of 0.7 and a MassBank weight of mz^2*I^0.5. Public libraries required a minimum signal threshold of 4, while in-house library required a threshold of 2, with a retention time tolerance of 0.5 min. The final feature list, including retention time, m/z, and peak area, was exported as a.csv file for further analysis. Missing values were imputed by the minimum observed value divided by 5.

#### Lentiviral Transduction and Selection of human induced pluripotent stem cells (hiPSCs) expressing P301L/V337M-mRuby

Human Induced pluripotent stem cells (iPSCs) derived from a familial Alzheimer's disease (fAD) patient (Coriell Catalog ID: AG25367) were obtained from the Coriell Institute for Medical Research. iPSCs were transduced with a lentiviral vector encoding the tau P301L/V337M double mutant fused to mRuby (Addgene plasmid #133057) to generate iPSCs stably expressing the P301L/V337M-mRuby fusion protein. For transduction, iPSCs were dissociated into single cells using ReLeSR and maintained in suspension. Lentiviral particles were added directly to the single-cell suspension at an appropriate multiplicity of infection (MOI), and the mixture was seeded onto Matrigel-coated plates. Cells were incubated for 24 h at 37 °C before the medium was replaced with fresh mTeSR Plus. Cells were cultured under standard conditions (37 °C, 5% CO₂) until they reached approximately 70% confluency. At this point, the cells were dissociated into single cells and subjected to fluorescence-activated cell sorting (FACS) using a BD AriaIII cell sorter. mRuby-positive cells were isolated based on fluorescence signal intensity. The sorted cells were then expanded and maintained for downstream applications.

#### Generation of neural progenitor cell (NPCs) from human induced pluripotent stem cells (hiPSCs) expressing P301L/V337M-mRuby

Human induced pluripotent stem cells (hiPSCs) stably expressing the P301L/V337M tau mutant fused to mRuby (see Lentiviral Transduction section; Addgene #133057) were cultured on 6-well plates (30006, SPL) pre-coated with Matrigel hESC-qualified Matrix (354277, Corning) in mTeSR Plus medium (ST05825, Stemcell Technologies). Upon reaching 70–80% confluency, cells were detached using ReLeSR (ST05872, Stemcell Technologies) and dissociated into single cells in mTeSR supplemented with 20 μM Y-27632 (ST72304, Stemcell Technologies). Cells were transduced with lentiviral vectors encoding NGN2 (79049, Addgene) and rtTA (19780, Addgene), and incubated for 4–6 h. The medium was then replaced with induction base medium composed of DMEM/F-12 (10565018, Gibco) and N2 supplement (100 ×; 17502048, Gibco), further supplemented with 2 μg/mL doxycycline hyclate (D9891, Sigma), 200 nM LDN193189 (6035, Tocris), 10 μM SB431542 (1614, Tocris), and 2 μM XAV939 (X3004, Sigma). After 24 h, cells were washed with DPBS (14190136, Thermo Fisher), and the same medium was reapplied with 5 μg/mL puromycin (P8833, Sigma) for 12 h to select infected cells. The medium was then refreshed following a DPBS wash.

To expand NPCs, cells were dissociated using Accutase (A6964, Sigma) in the presence of 10 μM Y-27632 and seeded at a density of 1.25 × 10^5^ cells/cm^2^ in DMEM/F-12 supplemented with 1% penicillin–streptomycin (P4333, Merck), MEM non-essential amino acids (11140–050, Thermo Fisher), B27 supplement minus vitamin A (50 ×; 12587010, Gibco), 10 ng/mL EGF (AF-100–15, Peprotech), 10 ng/mL bFGF (233-FB-025, R&D Systems), and 10 μM Y-27632. The following day, medium was changed to the same formulation without Y-27632, and NPCs were maintained under standard conditions for downstream applications.

#### Generation of induced neurons (iNs) from neural progenitor cells (NPCs)

Induced neurons (iNs) were generated from Neural progenitor cells (NPCs), by doxycycline-inducible NGN2-based neuronal programming, adapted from established protocol [37–40]. NPCs, derived as described above, were seeded onto 12-well plates (30012, SPL) pre-coated with Geltrex matrix (A1413302, Thermo Fisher Scientific) at a density of 1.2 × 10^5^ cells per well. Cells were plated in Neurobasal medium (21103049, Thermo Fisher Scientific) supplemented with B-27 Supplement Minus Vitamin A (50 ×; 12587010, Gibco), 10 ng/mL brain-derived neurotrophic factor (BDNF; 450–02, Peprotech), 10 ng/mL neurotrophin-3 (NT-3; 450–03, Peprotech). Y-27632 (10 μM; ST72304, Stemcell Technologies) was included in the medium on the day of seeding to promote cell survival, and was removed the following day by replacing with fresh medium lacking Y-27632. Cells were maintained in this neuronal differentiation medium for 14 days under standard culture conditions (37 °C, 5% CO₂) to allow maturation into iNs.

#### Immunocytochemistry

Induced neurons cultured on Geltrex-coated coverslips (A1413302, Thermo Fisher Scientific) were washed with phosphate-buffered saline (PBS) and fixed with 4% paraformaldehyde (PFA) for 20 min at room temperature. After PBS washes, cells were permeabilized and blocked for 1 h in PBS containing 0.3% Triton X-100 (X100, Sigma-Aldrich), 0.5 mg/mL bovine serum albumin (0332, VWR), and 5% normal horse serum (S-2000–20, Vector Laboratories). Cells were then incubated overnight at 4 °C with primary antibodies diluted in the same blocking buffer. Details of the primary antibodies are provided in Supplementary Table 2. The following day, cells were washed with PBS and incubated with Alexa Fluor–conjugated secondary antibodies (1:500) in PBS containing 5% normal serum for 1 h at room temperature. After additional PBS washes, nuclei were counterstained with DAPI (1:5000; D9542, Sigma-Aldrich) for 10 min. Coverslips were then mounted on glass slides, and fluorescence images were acquired using an LSM700 confocal microscope (Carl Zeiss) and ANDOR BC43 confocal microscopy system (Oxford Instruments).

#### Real-time quantitative-PCR (RT-qPCR)

Total RNA was isolated using the RNeasy Mini kit (74106, Qiagen) following the manufacturer's specifications. Subsequently, cDNA was synthesized at a concentration of 10 ng/μl using the Maxime RT PreMix kit (25081, Intronbio). Following cDNA synthesis, real-time qPCR was performed using the KAPA SYBR FAST qPCR kit (kk4602, KAPA Biosystems), and the primers used are listed in supplementary Table 4. The expression level of the target mRNA was analyzed using the ΔΔCt method, with the expression level of human GAPDH utilized as an endogenous control for normalization.

#### Western blot

Western blot was performed as previously described [41], with minor modifications.

Protein lysates were prepared from induced neurons (iNs) and mouse brain tissue using RIPA buffer supplemented with phenylmethanesulfonyl fluoride (PMSF; 93,482, Sigma-Aldrich), protein phosphatase inhibitor cocktails I and II (P-1517, P-1518, A.G. Scientific), and protease inhibitor cocktail (P8340, Sigma-Aldrich). Equal amounts of total protein (5–10 μg) were loaded onto 4–12% Bis–Tris polyacrylamide gels (Invitrogen) and electrophoresed in MES running buffer (NP0002, Pierce). Proteins were transferred to PVDF membranes (IPVH00010, Merck Millipore), which were then blocked in 5% skim milk dissolved in Tris-buffered saline with 0.1% Tween-20 (TBST) for 1 h at room temperature. Membranes were incubated overnight at 4 °C with primary antibodies diluted in blocking solution. A list of primary antibodies is provided in Supplementary Table 3. On the following day, membranes were washed with TBST and incubated for 1 h at room temperature with horseradish peroxidase (HRP)-conjugated secondary antibodies (1:2000) diluted in 2.5% skim milk. Immunoreactive bands were visualized using enhanced chemiluminescence (ECL) reagents and imaged with a LAS imaging system.

#### Study design, experimental unit, and replicates

All in vivo experiments were performed using mice as the experimental unit. Accordingly, n denotes the number of biologically independent mice included in each analysis (one mouse = one experimental unit). Where multiple measurements were obtained from the same mouse (e.g., multiple sections and/or microscopic fields), these measurements were averaged to yield a single value per mouse prior to statistical testing. Where applicable, N denotes the number of independent experimental repeats/cohorts performed on separate occasions (*N* = X), as indicated in the figure legends.

#### Statistical analysis

Data are presented as means ± standard errors of the mean (SEM). Statistical analyses were performed using GraphPad Prism 8 (GraphPad). Comparisons between two groups were performed using a two-tailed Student’s t-test. Comparisons among three or more groups were performed using one-way analysis of variance (ANOVA) followed by Tukey’s multiple-comparisons test, unless otherwise specified. *P* < 0.05 was considered as a statistically significant.

Bioinformatic Analysis of 16S rRNA Gene Amplicon Sequencing and Transcriptomic Data.

All statistical analyses were performed using raw data obtained from 16S rRNA gene amplicon sequencing and transcriptomic analysis. For group comparisons, one-way analysis of variance (ANOVA) was applied to normally distributed data, followed by Dunnett’s multiple comparisons test to compare each experimental group with the control group. When data did not meet normality assumptions, the Kruskal–Wallis test or two-tailed Mann–Whitney U test were used as non-parametric alternatives. Two-tailed simple linear regression analysis was used to evaluate correlations between gut microbial taxa abundance and other biomarkers. All statistical analyses were performed using GraphPad Prism software (version 10.4.1). A *p*-value less than 0.05 was considered statistically significant.

#### Metabolomics

Both two-group comparisons (Mann–Whitney U test) and PCA were conducted and visualized using the LMSstat package in R. Effect size (Hedge’s g) were calculated using effsize package (v0.8.1) in R. The metabolic network map was constructed by integrating biochemical pathway relationships (KEGG reactant pair database) and chemical structure similarity (Tanimoto score) and visualized by a group attribute layout using Cytoscape (v3.10.2) [42]. Correlation analyses were conducted based on Spearman’s correlation coefficients and visualized as heatmap using ComplexHeatmap package (v2.20.0) and chord diagram using circlize package (v 0.4.16) in R. Chemical classes were assigned to each metabolite using ClassyFire Batch (https://classyfire.wishartlab.com).

## Results

### SRK414 supplementation attenuates amyloid and tau pathology and improves cognitive performance in an Alzheimer’s disease model mice

**Fig. 1:**
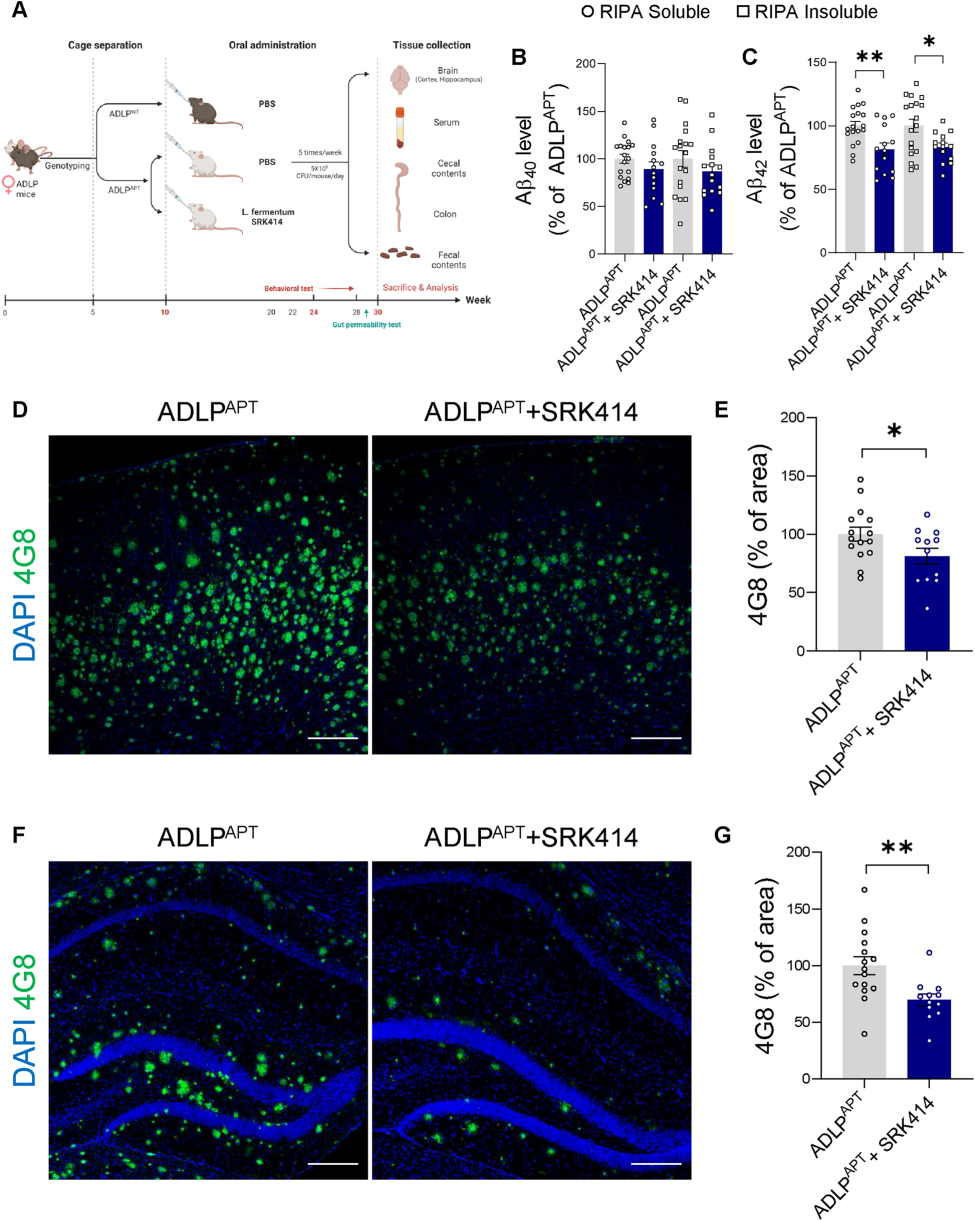
*L. fermentum* SRK414 reduces Aβ plaque burden in ADLP^APT^ mice. **A** Schematic of the experimental design to assess the effect of *L. fermentum* SRK414 in ADLP^APT^ mice. Female ADLP mice were genotyped at 5 weeks of age and separated into two groups based on genotype (ADLP^WT^ and ADLP^APT^). At 10 weeks of age, mice were orally administered either PBS or SRK414 (5 × 10^9^ CFU/mouse/day) five times per week until sacrifice. Behavioral testing was conducted at week 28, and gut permeability testing was performed at week 30. At 30 weeks, mice were sacrificed for tissue collection, including brain (cortex and hippocampus), serum, cecal contents, colon, and fecal contents for downstream analysis. Figure generated using Biorender. **B** and **C** Measurement of Aβ40 (**B**) and Aβ42 (**C**) in RIPA-soluble and insoluble fraction from the cortex, assessed by ELISA. Data are presented as mean ± SEM with each data point representing a mouse. Statistical significance was determined using a two-tailed unpaired t test. **D** Representative images of amyloid plaques (4G8) in the cortex of ADLP^APT^ mice or ADLP^APT^ + SRK414 mice. Scale bar, 200 μm. **E** Quantification of 4G8 signals. Data are presented as mean ± SEM with each data point representing a mouse. Statistical significance was determined using a two-tailed unpaired t test. **F** Representative images of amyloid plaques (4G8) in the hippocampus of ADLP^APT^ mice or ADLP^APT^ + SRK414 mice. Scale bar, 200 μm. **G** Quantification of 4G8 signals. Data are presented as mean ± SEM with each data point representing a mouse. Statistical significance was determined using a two-tailed unpaired t test. Sample size (biological replicates): (**B**-**C**), *n* = 18 per ADLP^APT^ mouse, *n* = 14 per ADLP^APT^ + SRK414 mouse; (**D-G** ), *n* = 15 per ADLP^APT^ mouse, *n* = 12 per ADLP^APT^ + SRK414 mouse). Independent experimental repeats: *N* = 2

AD is characterized by the accumulation of amyloid-beta (Aβ) plaques, and intracellular neurofibrillary tangles composed of hyperphosphorylated tau protein. In our previous study, fecal microbiota transplantation (FMT) from healthy wild-type (ADLP^WT^) mice alleviated AD-related pathology in ADLP^APT^ mice [13]. To test whether probiotics similarly ameliorate AD pathology, SRK414 was orally administered 5 days per week for five months, starting at 10 weeks of age, and its effects on brain pathology and gut-brain axis interactions were subsequently evaluated (Fig. 1A).

SRK414 treatment did not affect Aβ40 levels (Fig. 1B), but significantly reduced both soluble and insoluble Aβ42 levels in the cortex, as measured by ELISA (Fig. 1C). Immunohistochemistry further confirmed a marked reduction in Aβ plaque burden in both the cortex and hippocampus of SRK414-treated mice compared to untreated ADLP^APT^ controls (Fig. 1, D to G).

**Fig. 2:**
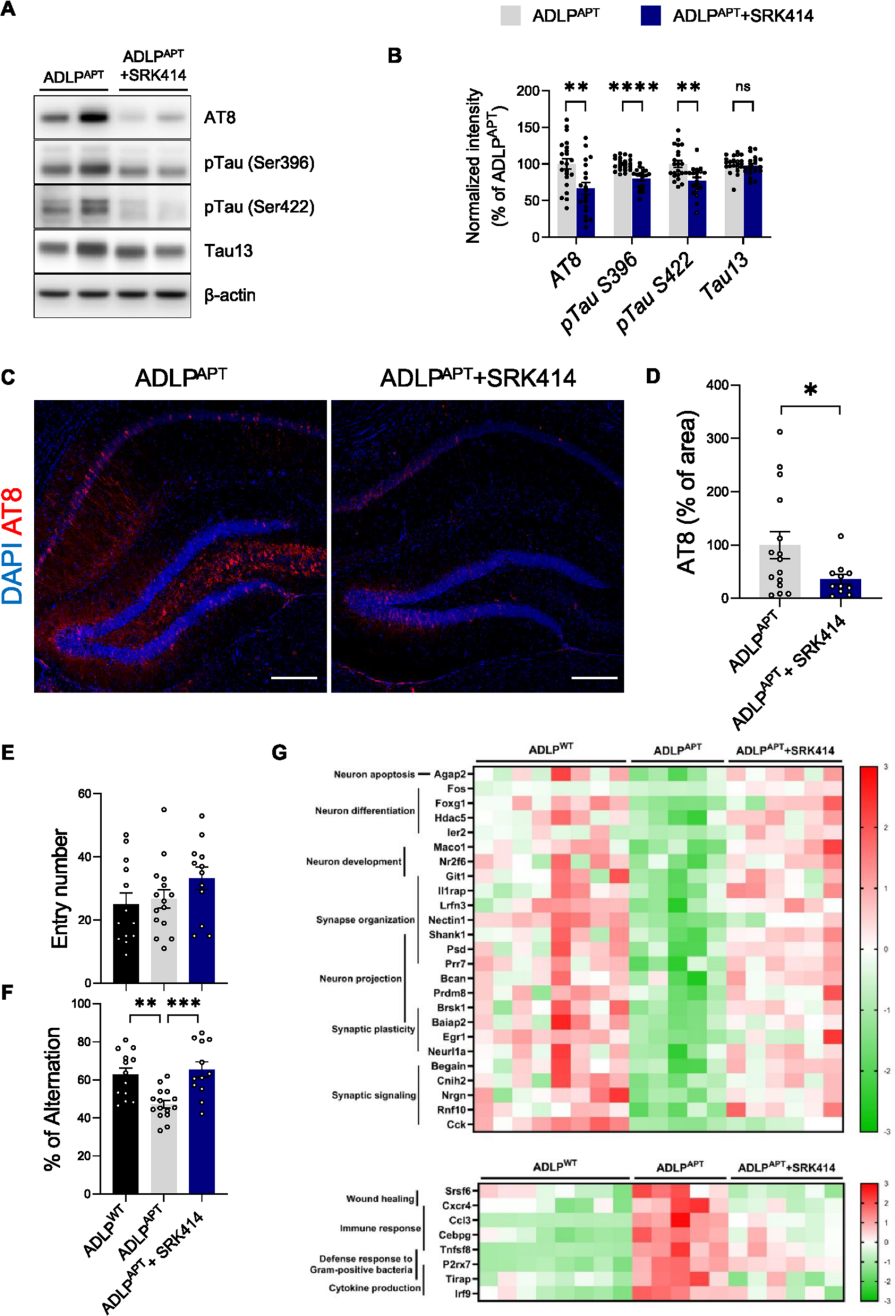
*L. fermentum* SRK414 reduces tau phosphorylation and improves memory deficits in ADLP^APT^ mice. **A-B** Representative immunoblots (**A**) and quantification of pTau396, pTau422, AT8 and Tau13 in Western blot (**B**). Data are presented as mean ± SEM with each data point representing a mouse. Statistical significance was determined using a two-tailed unpaired t test. **C** Representative images of AT8 in the hippocampus of ADLP^APT^ mice or ADLP^APT^ + SRK414 mice. Scale bar, 200 μm. **D** Quantification of AT8 signals. Data are presented as mean ± SEM with each data point representing a mouse. Statistical significance was determined using a two-tailed unpaired t test. **E** Entry number and (**F**) percentage of alternations assessed by Y-maze task. Data are presented as mean ± SEM with each data point representing a mouse. Statistical analysis was determined using one-way ANOVA followed by Tukey’s multiple comparisons test. **G** Representative heatmap of differentially expressed genes (DEGs) showing gene expression changes in the hippocampus. Sample size (biological replicates): (**A**-**B),** *n* = 22 per ADLP^APT^ mouse, *n* = 19 per ADLP^APT^+ SRK414 mouse; (**C**-**D),** *n* = 15 per ADLP^APT^ mouse, *n* = 12 per ADLP^APT^ + SRK414 mouse; (**E**–**F)**, *n* = 13 per ADLP^WT^ mouse, *n* = 15 per ADLP^APT^ mouse, *n* = 12 per ADLP^APT^ + SRK414 mouse). Independent experimental repeats: (**B**, *N* = 3; **C**-**F**, *N* = 2)

Tau pathology was also evaluated in the hippocampal tissues (Fig. 2A). Western blot analysis revealed a significant decrease in tau phosphorylation at pathological sites associated with paired helical filaments (PHFs) and neurofibrillary tangles (NFTs), including Ser202/Thr205 (AT8), Ser396, and Ser422 [43] (Fig. 2B). Additionally, AT8 immunostaining confirmed reduced AT8-positive signal throughout the hippocampus in SRK414-treated ADLP^APT^ mice compared to untreated ADLP^APT^ mice (Fig. 2, C and D).

Cognitive performance assessed by Y-maze showed improved spatial short-term memory in ADLP^APT^ following SRK414 treatment (Fig. 2F), without differences in total arm entries (Fig. 2E), body weight (fig. S1A), or locomotor activity in the open field test (fig. S1B), suggesting that cognitive improvements were not due to changes in mobility or general health. Consistently, SRK414-treated ADLP^APT^ mice also exhibited increased novel object preference in the novel object recognition (NOR) test (fig. S1C), indicating improved object recognition memory.

Collectively, these results suggest that SRK414 ameliorates Aβ and tau pathologies and improves cognitive performance in ADLP^APT^ mice, likely through modulation of the gut-brain axis.

### SRK414 normalizes transcriptional signatures linked to synaptic plasticity and mitochondrial function in Alzheimer’s disease

To investigate the molecular mechanisms underlying SRK414’s therapeutic effects, we performed transcriptomic profiling of brain tissues from ADLP^WT^, ADLP^APT^, and SRK414-treated ADLP^APT^ mice. Differentially expressed gene (DEG) analysis revealed widespread transcriptional dysregulation in the hippocampus and cortex of ADLP^APT^ mice compared to ADLP^WT^ controls, which was substantially restored following SRK414 treatment.

In the hippocampus, 222 genes were upregulated and 285 downregulated in SRK414-treated ADLP^APT^ mice compared to untreated ADLP^APT^ mice (fig. S2A). Notably, genes associated with neuronal development, synaptic structure, plasticity, and neurotransmission—suppressed in ADLP^APT^ mice—were transcriptionally reactivated following SRK414 treatment (Fig. 2G).

Immediate early genes (IEGs) such as *Fos* and *Jun *[44], as well as the neuroactive hormone gene *Prl* (prolactin) [45] were upregulated in both the cortex and hippocampus after SRK414 treatment (fig. S2, B and C). IEGs regulate synaptic plasticity and memory [46, 47], and prolactin has been shown to promote neurogenesis and synaptogenesis, and to protect hippocampal neurons from chronic stress [48, 49]. These changes suggest that SRK414 activates transcriptional programs linked to cognitive function and neuronal resilience. To explore protein-level functional interactions associated with SRK414-induced gene expression, a protein–protein interaction (PPI) network analysis was conducted. Enriched biological processes included transcriptional regulation via RNA polymerase II, oxygen sensing, calcium signaling, and nervous system activity, supporting a broad neuronal homeostatic effect (fig. S2, D to E).

In parallel with neuronal reprogramming, the upregulation of immune response genes observed in ADLP^APT^ mice, particularly genes involved in wound healing, cytokine-mediated signaling pathways, and antibacterial defense mechanisms, was significantly attenuated following administration of SRK414 (Fig. 2G). These findings were accompanied by reduced Iba1-positive microglial activation in the hippocampus, indicating suppression of chronic neuroinflammation (fig. S2, F and G).

In the cortex, 173 genes were upregulated and 301 downregulated by SRK414 treatment (fig. S3A). Gene set enrichment analysis (GSEA) revealed significant activation of oxidative phosphorylation pathways (fig. S3B), with transcriptional upregulation of multiple mitochondrial electron transport chain components, including NADH dehydrogenase subunits (*Ndufa2*, *Ndufa5*, *Ndufb7*, *Ndufs7*, *Ndufs8*), succinate dehydrogenase (*Sdhd*), cytochrome c oxidase/reductase genes (*Cox10*, *Cox5b*, *Cox6a1*, *Cox7c*, *Cox8a*, *Uqcrq*), and ATP synthase subunits (*Atp5d*, *Atp5f2*) (fig. S3, C to F). These results suggest improved bioenergetic capacity and ATP synthesis in SRK414-treated cortical tissue.

Together, these transcriptomic findings demonstrate that SRK414 modulates gene networks related to neuronal plasticity, mitochondrial metabolism, and immune regulation. SRK414 thereby exerts multifaceted neuroprotective effects, supporting its potential as a therapeutic candidate for AD.

### SRK414 reshapes gut microbial composition and diversity in ADLP^APT^ mice

**Fig. 3:**
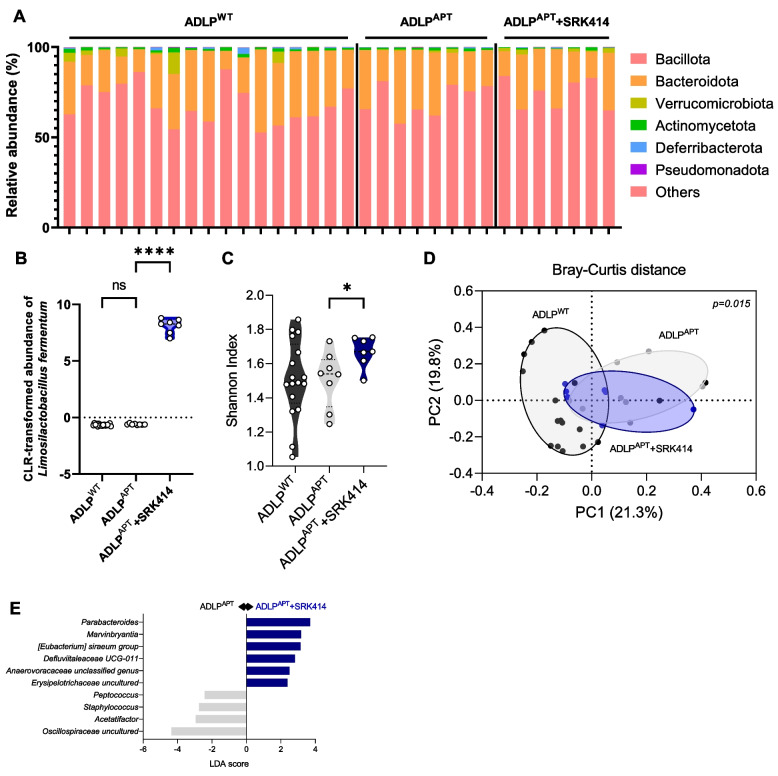
*L. fermentum* SRK414 modulated gut microbiota composition and diversity in ADLP^APT^ mice. **A** Taxonomic composition of the gut microbiota at the phylum level. **B** Centered log-ratio (CLR)-transformed abundance of *Limosilactobacillus fermentum* in cecal contents. Violin plots represent data distribution with individual data points shown. Statistical analysis was performed using one-way analysis of variance (ANOVA) followed by Dunnett’s multiple comparisons test. *p* < 0.05 was considered statistically significant. **C** Alpha diversity assessed by Shannon’s index. Violin plots represent the distribution of data with individual values indicated. Statistical analysis was performed using the nonparametric Mann–Whitney U test. *p* < 0.05 was considered statistically significant. **D** Beta diversity visualized by Principal Coordinates Analysis (PCoA) based on Bray–Curtis dissimilarity. **E** Differentially abundant taxa identified by Linear discriminant analysis Effect Size (LEfSe). Sample size (biological replicates): (**A**-**E)**, *n* = 17 per ADLP^WT^ mouse, *n* = 8 per ADLP^APT^ mouse, *n* = 7 per ADLP^APT^ + SRK414 mouse. Independent experimental repeats: *N* = 1

To investigate the impact of SRK414 on gut–brain axis regulation in the ADLP model, the composition of the cecal microbiota and its association with physiological parameters were analyzed. SRK414 treatment induced compositional shifts in the gut microbiota at the phylum level and significantly increased the Centered log-ratio (CLR)-transformed abundance of *Limosilactobacillus fermentum* in the SRK414-treated ADLP^APT^ mice compared to untreated ADLP^APT^ mice (*p* < *0.001*) (Fig. 3, A and B).

Alpha diversity, assessed using Shannon’s index, was significantly reduced in the ADLP^APT^ mice compared to ADLP^WT^ mice, whereas SRK414 treatment led to a notable increase in alpha diversity compared to the ADLP^APT^ group (*p* = *0.029*) (Fig. 3C). Beta diversity using Principal Coordinates Analysis (PCoA) based on Bray–Curtis distance revealed a distinct clustering pattern of microbial communities among the ADLP^WT^, ADLP^APT^, and SRK414-treated groups (*p* = *0.015*) (Fig. 3D).

At the genus level, linear discriminant analysis (LDA) identified specific taxonomic differences. The relative abundance of *Parabacteroides*, *Marvinbryantia*, *Eubacterium*, *Defluviitaleaceae* UCG-011, *Anaerovoracaceae* unclassified genus, and *Erysipelotrichaceae* uncultured was elevated in the SRK414-treated ADLP^APT^ group, while *Oscillospiraceae* uncultured*, Acetatifactor*, *Staphylococcus*, and *Peptococcus*, were predominantly enriched in the ADLP^APT^ group (Fig. 3E).

### Restoration of intestinal barrier integrity suppresses antigen presentation and gut immune activation

**Fig. 4:**
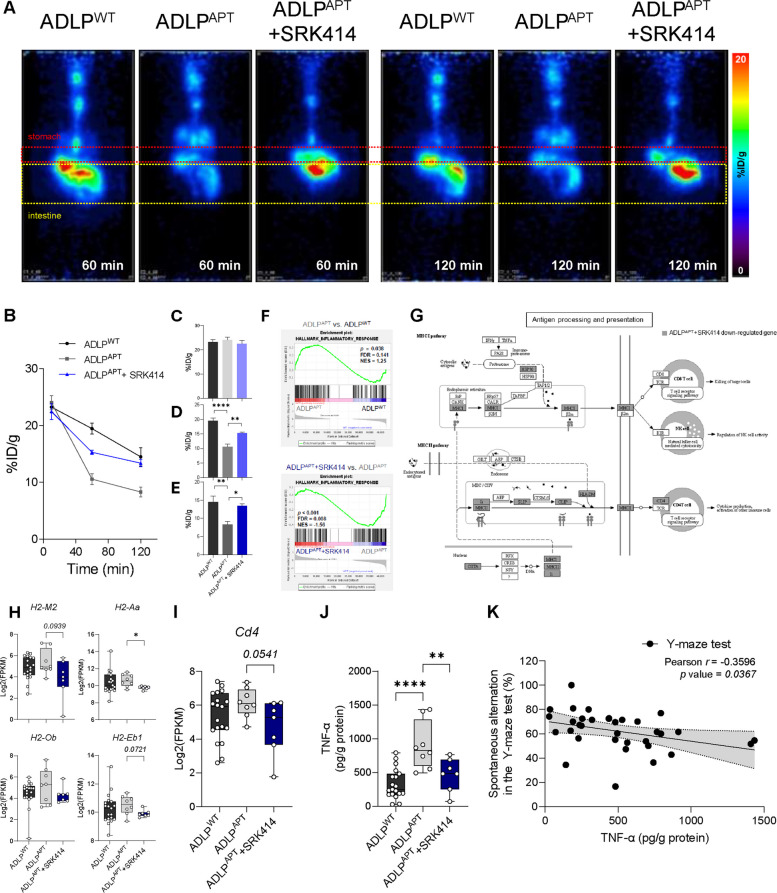
*L. fermentum* SRK414 improves gut barrier integrity and reduces colonic inflammation in ADLP^APT^ mice. **A** Representative in vivo images of FITC–dextran retention in the gastrointestinal tract at 60 and 120 min after oral administration. Regions of interest (ROIs) for the stomach (red box) and intestine (yellow box) are indicated. **B** Quantitative analysis of FITC–dextran fluorescence over time. **C–E** Quantification of integrated signal intensity in the intestinal ROI at 10 (**C**), 60 (**D**) and 120 min (**E**). Data are presented as mean ± SEM with each data point representing a mouse. Statistical analysis was determined using one-way ANOVA followed by Tukey’s multiple comparisons test. **F** Gene Set Enrichment Analysis (GSEA) enrichment plot of the colon transcriptome. **G** Downregulated genes involved in antigen processing and presentation. **H** mRNA expression levels of *H2-M2*, *H2-Aa*, *H2-Ob*, and *H2-Eb1* in colon tissue. Box plots display the full data range (minimum to maximum) along with individual data points. Statistical analysis was performed using the nonparametric Mann–Whitney U test. *p* < 0.05 was considered statistically significant. **I** Colonic *Cd4* mRNA expression levels. Box plots display the full data range (minimum to maximum) along with individual data points. Statistical analysis was performed using the nonparametric Mann–Whitney U test. *p* < 0.05 was considered statistically significant. **J** TNF-α protein concentration in colon. Box plots display the full data range (minimum to maximum) along with individual data points. Statistical analysis was performed using one-way analysis of variance (ANOVA) followed by Dunnett’s multiple comparisons test. *p* < 0.05 was considered statistically significant. **K** Correlation analysis between colonic TNF-α protein concentration and spontaneous alternation behavior in the Y-maze test. Scatter plots depict the correlation between colonic TNF-α protein concentration and spontaneous alternation behavior with a simple linear regression line overlaid. Pearson’s correlation coefficient (R) was used to assess the strength and direction of the linear relationship. *p* < 0.05 was considered statistically significant. Sample size (biological replicates): (**A**-**E**), *n* = 5 per ADLP^WT^ mouse, *n* = 5 per ADLP^APT^ mouse, *n* = 4 per ADLP^APT^ + SRK414 mouse; (**H**–**K)**, *n* = 13 per ADLP^WT^ mouse, *n* = 15 per ADLP^APT^ mouse, *n* = 12 per ADLP^APT^ + SRK414 mouse. Independent experimental repeats: (**A**-**E**, *N* = 1; **H–K**, *N* = 1)

Gut inflammation and altered gut microbiota composition are increasingly recognized as key contributors to the pathogenesis of AD and are linked to gut barrier dysfunction [50]. In our previous study, we demonstrated that 7-month-old ADLP^APT^ mice exhibited high gut permeability and increased gut inflammation [13]. To further evaluate intestinal barrier function, we performed in vivo imaging using FITC–dextran (4 kDa) conjugated with a radiotracer. Following oral administration, the intestinal retention of the labeled dextran was visualized and quantified. Because FITC–dextran translocation into circulation reflects barrier leakage, a higher intestinal signal intensity corresponds to lower permeability and improved barrier integrity. Consistent with our earlier findings, ADLP^APT^ mice showed a rapid decline in intestinal FITC–dextran signal over time compared to ADLP^WT^ controls, indicative of increased intestinal permeability and compromised epithelial integrity. In contrast, SRK414-treated ADLP^APT^ mice displayed significantly greater intestinal retention of FITC–dextran, comparable to the levels observed in ADLP^WT^ controls (Fig. 4, A and B). Quantitative analysis showed that immediately after administration of radiolabeled FITC–dextran, all groups exhibited comparable signal intensities (Fig. 4C). However, at 60- and 120-min post-administration, SRK414-treated ADLP^APT^ mice maintained significantly higher intestinal signal intensity compared to untreated ADLP^APT^ mice, indicating that SRK414 improved intestinal barrier integrity (Fig. 4, D and E). Consistent with the imaging-based retention readout, SRK414-treated ADLP^APT^ mice exhibited significantly lower serum FITC–dextran fluorescence compared with untreated ADLP^APT^ controls (fig. S4A), supporting improved epithelial barrier integrity. To further examine whether the barrier-restorative effect depended on bacterial viability, we used iPSC derived apical-out colon organoids pre-exposed to TNF-α and IFN-γ to induce barrier dysfunction. Live SRK414 significantly reduced the FITC-dextran intensity within the organoid interior, whereas heat-killed SRK414 showed only a modest, non-significant trend toward reduction (fig. S4, B and C). These findings suggest that bacterial viability contributes to the barrier-protective effect observed in this organoid system.

To investigate gut inflammation at the molecular level, RNA sequencing was performed on colon tissues from ADLP^WT^, ADLP^APT^, and SRK414-treated ADLP^APT^ groups. Differential gene expression analysis revealed distinct transcriptomic signatures among the groups, highlighting inflammation-associated transcriptional changes in the ADLP^APT^ mice. Notably, the administration of SRK414 significantly downregulated genes associated with inflammatory response pathways, as shown in the enrichment plot (Fig. 4F). In particular, genes involved in the antigen processing and presentation pathway—including MHC class I, MHC class II, HSP70, SLIP, and CLIP—were markedly suppressed in the SRK414-treated ADLP^APT^ group (Fig. 4G). Key MHC-related genes such as *H2-M2*, *H2-Aa*, *H2-Ob*, and *H2-Eb1* showed lower expression in the SRK414-treated ADLP^APT^ mice compared to ADLP^APT^ (Fig. 4H). Furthermore, expression of *Cd4*, a critical marker for T cell–mediated immune activation [51], was reduced following SRK414 treatment (Fig.  4I), supporting the immunomodulatory and anti-inflammatory effects of SRK414 in the gut.

Consistent with transcriptomic findings, colonic levels of pro-inflammatory cytokine TNF-α were significantly elevated in ADLP^APT^ mice relative to ADLP^WT^, in line with prior reports of increased intestinal, colonic TNF-α in AD-relevant mouse models [32, 52, 53]. Importantly, SRK414 treatment markedly decreased TNF-α protein levels in the colon (Fig. 4J). Moreover, TNF-α concentrations were negatively correlated with cognitive performance in the Y-maze test (Pearson r = –0.3596, *p* = *0.0367*) (Fig. 4K), consistent with prior literature linking elevated TNF-α, systemic inflammatory burden to accelerated cognitive decline [54].

Collectively, these results indicate that SRK414 improves ADLP^APT^-induced intestinal barrier dysfunction and suppresses gut inflammation, underscoring its therapeutic potential in modulating gastrointestinal homeostasis alongside its neuroprotective effects.

### SRK414 is associated with a shift in hippocampal metabolic profiles and coordinated tryptophan-kynurenine pathway changes

**Fig. 5:**
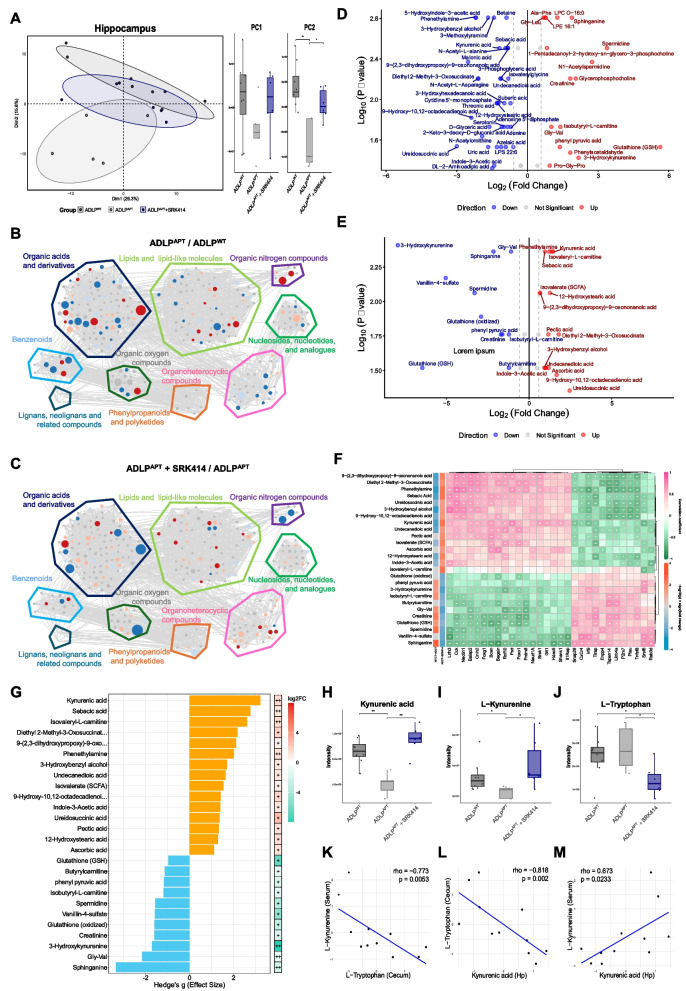
*L. fermentum* SRK414 improves hippocampal metabolic profiles in ADLP^APT^ mice. **A** PCA score plot and box plots of PC1 and PC2 for hippocampal samples. Asterisks indicate statistical significance (* *P* < 0.05, ** *P* < 0.01; Mann–Whitney U test). **B–C** Metabolic networks showing altered metabolite modules due to AD pathology (**B**) and SRK414 treatment (**C**). Statistical significance was determined by the Mann–Whitney U test. Node color reflects effect size and significance (sky blue: Hedge’s g ≤ –0.8; blue: Hedge’s g ≤ –0.8, *P* < 0.05; orange: Hedge’s g ≥ 0.8; red: Hedge’s g ≥ 0.8, *P* < 0.05); node size indicates fold change magnitude. **D–E** Volcano plots of hippocampal metabolites altered by AD pathology (**D**) and SRK414 treatment (**E**). Statistical significance was determined by the Mann–Whitney U test. Dots with *P* < 0.05 are shown; red indicates upregulation (fold change ≥ 1.5), blue indicates downregulation (fold change ≤ –1.5). Fold changes were calculated as ADLP^APT^ over ADLP^WT^ and ADLP^APT^ + SRK414 over ADLP^APT^. **F** Spearman correlation between significantly altered hippocampal metabolites and normalized transcripts. Color scale indicates correlation (pink: positive; green: negative); asterisks denote significance (* *P* < 0.05, ** *P* < 0.01). Side heatmap shows direction of change (–log10 *P* × sign fold change): red (increased), blue (decreased), grey (no change). **G** Metabolites ranked by effect size (|Hedge’s g|≥ 0.8) in response to SRK414. Orange and blue bars indicate increased or decreased effect sizes, respectively. The annotation bar shows fold change (log₂ scale), with red for upregulation and green for downregulation. Significance is marked (+ *P* < 0.05, + + *P* < 0.01; Mann–Whitney U test). **H–J** Box plots of representative metabolites: **H** kynurenic acid (KYNA, hippocampus), (**I**) kynurenine (KYN, serum), **J** tryptophan (TRP, cecum). Asterisks indicate statistical significance (* *P* < 0.05, ** *P* < 0.01; Mann–Whitney U test). **K–M** Correlation scatter plots of tryptophan–kynurenine pathway metabolites across the cecum–serum–hippocampus axis under SRK414: **(K)** tryptophan (TRP, cecum) vs. kynurenine (KYN, serum), (**L**) tryptophan (TRP, cecum) vs. kynurenic acid (KYNA, hippocampus), (**M**) kynurenine (KYN, serum) vs. kynurenic acid (KYNA, hippocampus). Statistical significance was determined by spearman correlation. Sample size (biological replicates): (**A**-**M)**, *n* = 8 per ADLP^WT^ mouse, *n* = 5 per ADLP^APT^ mouse, *n* = 6 per ADLP^APT^ + SRK414 mouse. Independent experimental repeats: *N* = 1

We next performed integrative metabolomic analysis across multiple tissues to investigate whether these gut-mediated effects extend to metabolic regulation in the brain. Principal component analysis (PCA) was conducted to evaluate the effect of the SRK414 treatment on integrative metabolic profiles. In the hippocampus, the SRK414-treated ADLP^APT^ group (ADLP^APT^ + SRK414) clustered closely with the ADLP^WT^ group, while being distinct from the ADLP^APT^ group (Fig. 5A). In contrast, remaining biological samples (cortex, serum, cecum, and feces) did not show the normalized profiles following SRK414 treatment (fig. S5, A to D).

To characterize global metabolic alterations in ADLP^APT^ mice and evaluate the effects of SRK414 treatment, we applied metabolic network analysis using MetaMapp framework, which integrates chemical similarity (Tanimoto scores) and biochemical connectivity (KEGG reactant pairs), to provide a module-level overview of metabolite perturbations [55]. Disease-induced metabolic changes were observed across multiple interconnected metabolic modules, indicating systemic dysregulation of functionally linked biochemical pathways (Fig. 5B). SRK414 treatment led to marked restoration of organoheterocyclic compounds, organic nitrogen compounds and organic oxygen compounds to levels similar to those in ADLP^WT^ mice (Fig. 5C). Notably, kynurenic acid (KYNA), sebacic acid, isovaleryl-L-carnitine, and phenethylamine showed the most substantial increases (Mann–Whitney U-test *p* < *0.005),* while 3-hydroxykynurenine (3-OHKYN), sphinganine and Gly-Val were significantly decreased (Mann–Whitney U-test *p* < *0.005*). All these compounds, except isovaleryl-L-carnitine, were restored to levels similar to those in ADLP^WT^ mice following SRK414 treatment, suggesting that the treatment promotes metabolic recovery (Fig. 5, D and E). Particularly, the elevated levels of sebacic acid, isovaleryl-L-carnitine, isovalerate and 12-hydroxystearic acid, suggest enhanced lipid metabolism, including increased beta-oxidation and mitochondrial energy turnover (Fig. 5E). Additionally, hippocampal levels of indole-3-acetic acid were elevated, accompanied by an increase in its serum precursor, indolelactic acid (fig. S5, E and F), implying a systemic shift in tryptophan metabolism, with both central and peripheral modulation.

Metabolites significantly altered by SRK414 treatment (*p* < *0.05*, |fold change|≥ 1.5), also showed strong correlations with normalized transcriptomic profiles related to neural precursor cell proliferation, neuron projection development, synapse organization, synaptic signaling, wound healing, neutrophil degranulation, cytokine regulation, and defense responses to gram-positive bacteria (Fig. 5F). To prioritize key metabolites underlying therapeutic effect of SRK414, we ranked significantly altered metabolites using the effect size criterion (|hedge's g|≥ 0.8). KYNA exhibited the largest positive effect size in response to SRK414 treatment (effect size = 3.23), followed by sebacic acid and isovaleryl-carnitine. In contrast, sphinganine showed the largest negative effect size (effect size = –3.34), followed by Gly-Val and 3-hydroxykynurenine (3-OHKYN) (Fig. 5G).

To further examine inter-organ metabolic coordination, we performed correlation analysis among significantly altered metabolites in the hippocampus, serum, and cecum (fig. S5G). The chord diagram highlighted complex inter-organ connectivity, indicating active metabolic communication between central and peripheral compartments. Of note, KYNA in the hippocampus was strongly inter-correlated with its precursor, kynurenine (KYN) in the serum and the upstream metabolite, tryptophan (TRP) in the cecum (cecum-serum: rho = −0.773, p = 0.0053, cecum-hippocampus: rho = −0.818, *p* = *0.002*, hippocampus-serum: rho = 0.673, *p* = *0.0233*) (Fig. 5, H to M). Consistent with these findings, the increased serum KYN/TRP ratio, a commonly used marker of kynurenine pathway activation [56, 57], supports systemic engagement of the kynurenine pathway (fig. S5H). At the hippocampal level, 3-OHKYN, a neurotoxic kynurenine-pathway metabolite, was decreased and showed a significant negative correlation with KYNA (rho = −0.818, *p* = *0.0021*) (fig. S5, I and J)*.* The resulting elevation in the KYNA/3-OHKYN ratio supports a relative metabolic shift in kynurenine-pathway toward the KYNA-associated neuroprotective branch (fig. S5, K to M). These coordinated changes are consistent with cross-compartment coupling across the cecum, serum, hippocampus along the tryptophan-kynurenine-kynurenic acid metabolic axis.

Together, these findings indicate that SRK414 treatment mitigates hippocampal metabolic dysregulation, particularly by tryptophan-related metabolism and lipid metabolic remodeling, accompanied by normalization of transcriptomic profiles. This metabolic modulation may represent a complementary molecular mechanism, or at least complementary mechanism associated with recovery-related changes.

### Kynurenic acid attenuates tau-related and inflammatory readouts and modulates lipid droplet–associated dysregulation

**Fig. 6:**
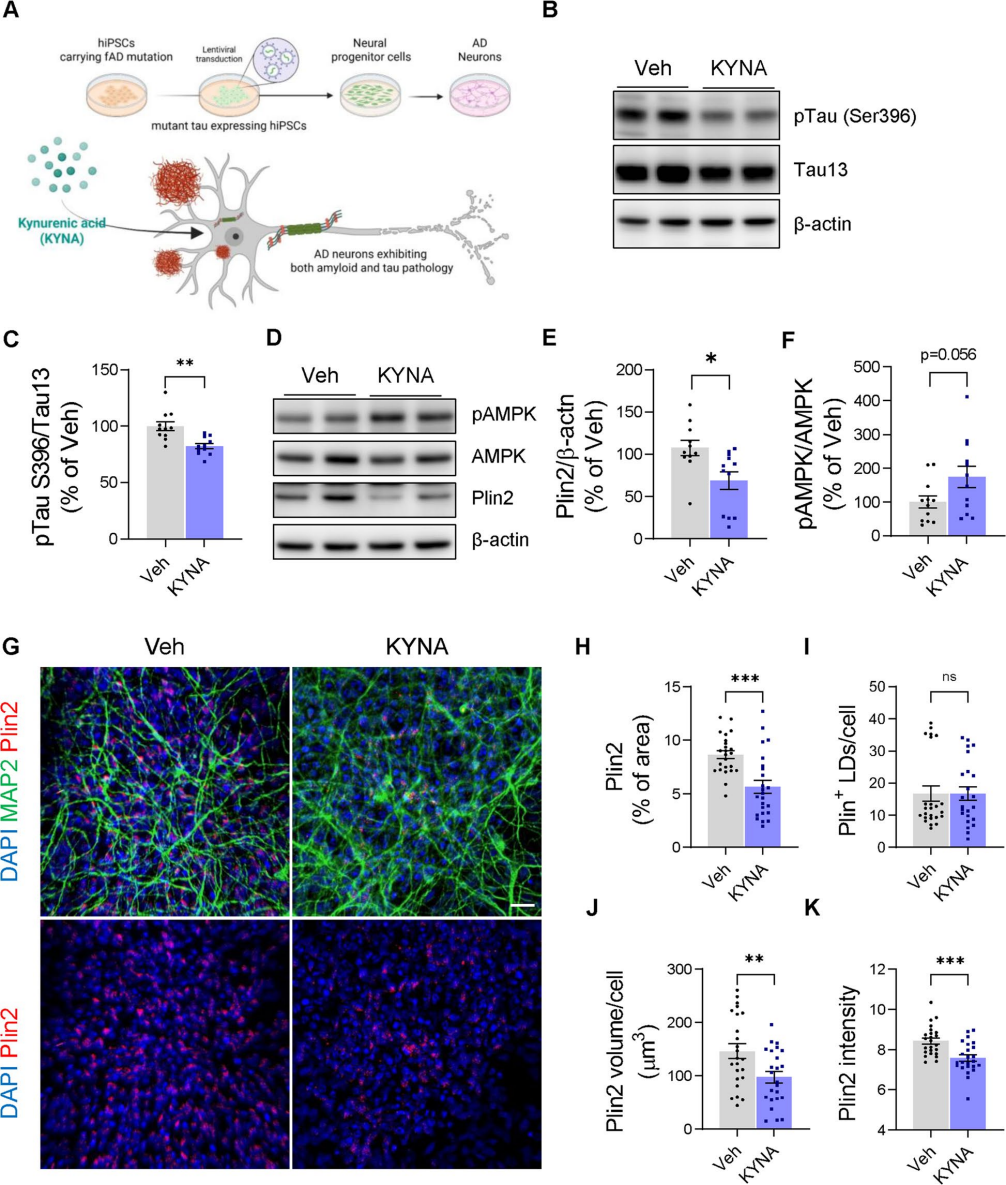
KYNA treatment reduces tau phosphorylation and lipid droplet accumulation in AD patient-derived neurons. **A** Schematic representation of the differentiation of patient-derived iPSCs into AD neurons, followed by KYNA treatment (50 μM, 24 h). Figure generated using Biorender. **B–C** Representative immunoblots (**B**) and quantification (**C**) of pTau S396/Tau13 in AD neurons treated with Veh or KYNA (50 μM, 24 h). Data are presented as mean ± SEM. Statistical significance was determined using a two-tailed unpaired t test. **D–F** Representative immunoblots (**D**) and quantification of Plin2/β-actin (**E**) and pAMPK/AMPK (**F**) in AD neurons treated with Veh or KYNA (50 μM, 24 h). Data are presented as mean ± SEM. Statistical significance was determined using a two-tailed unpaired t test. **G** Representative images of lipid droplets (Plin2) and MAP2^+^ in AD neurons. **H–K** Quantification of Plin2 area fraction (**H**), the number of Plin2.^+^ LDs per cell (**I**), total Plin2 volume per cell (**J**) and Plin2 mean intensity (**K**) in AD neurons treated with Veh or KYNA (50 μM, 24 h). Data are presented as mean ± SEM. Statistical significance was determined using a two-tailed unpaired t test. Sample size (biological replicates): (**B**,**C),***n* = 11 per group; (**D**,**F),***n* = 12 per group. (**G**-**K**), *n* = 24 per group. Independent experimental repeats: (**B**-**F**, *N* = 2; **G-K**, *N* = 1)

To functionally validate the metabolic alteration induced by SRK414, we examined the effects of KYNA, the metabolite with the largest effect size by treatment, in disease-relevant cellular models. In AD neurons exhibiting both amyloid and tau pathologies (Fig. 6A), KYNA treatment significantly reduced tau phosphorylation (Fig. 6, B and C), supporting its neuroprotective role. Similarly, in primary mouse microglia stimulated with Aβ, KYNA markedly suppressed the expression of pro-inflammatory cytokines, including TNF-α, IL-1β, and IL-18 (fig. S6, A to D) suggesting anti-inflammatory effects of KYNA on innate immune cells.

Given the enhancement of lipid metabolism observed in metabolomic analysis, we further assessed lipid droplet dynamics in the hippocampus. SRK414-treated ADLP^APT^ mice exhibited a reduction in lipid droplet accumulation compared to untreated controls (fig. S6, E and F). To determine whether KYNA directly modulates lipid metabolism, we treated AD neurons with KYNA and assessed AMPK activation and the expression of Plin2, a major lipid droplet-associated protein. Western blot analysis revealed increased phosphorylation of AMPK and a notable decrease in Plin2 expression, following KYNA treatment (Fig. 6, D to F).

Immunofluorescence analysis further confirmed that the area fraction of Plin2 was decreased in KYNA-treated AD patient-derived neurons compared to untreated neurons (Fig. 6, G and H). While the number of Plin2-positive lipid droplets remained unchanged (Fig. 6I), KYNA treatment significantly reduced the volume and intensity of Plin2-positive lipid structures (Fig. 6, J and K), indicating that KYNA selectively remodels pathological lipid droplets without disrupting physiological lipid storage, thereby restoring lipid metabolic balance.

Collectively, these results demonstrate that SRK414 supplementation is accompanied by an increase in KYNA and a shift in hippocampal metabolic profiles, alongside reduced tau pathology, suppressed neuroinflammation, and improved lipid homeostasis. Notably, KYNA treatment recapitulated key phenotypes in vitro. Specifically, KYNA attenuated tau-related readouts and lipid droplet accumulation in induced neurons and promoted anti-inflammatory responses in primary microglia. These findings support a contributory role for KYNA in the observed protective effects, although direct causal involvement in vivo remains to be established.

## Discussion

Alzheimer’s disease (AD) emerges from the complex interplay of Aβ and tau aggregation, neuroinflammation, and metabolic dysregulation [58]. Recognizing this multifactorial nature of AD, recent studies have increasingly focused on the gut microbiota as a potential modulator of brain health. While previous studies, including our own, have demonstrated that fecal microbiota transplantation (FMT) from healthy donors can mitigate AD-related pathology in mouse models [13, 59], mechanisms by which defined probiotic strains influence neurodegeneration remain unclear.

Here, we show that SRK414 ameliorates cognitive decline and hallmark AD pathologies in the ADLP^APT^ mouse by modulating the gut–brain axis. SRK414 reduced Aβ plaque burden and tau hyperphosphorylation, improved spatial memory, remodeled brain transcriptomic profiles, and restored gut homeostasis, including microbial composition, intestinal inflammation, and barrier integrity. Notably, colon inflammation levels negatively correlated with cognitive performance, underscoring the relevance of the gut–brain axis in AD pathogenesis.

To better elucidate the mechanisms linking gut microbial remodeling to brain pathology, we conducted multi-compartment metabolomic profiling. Principal component analysis revealed that the hippocampal metabolic profile in the SRK414-treated group aligned closely with the profile of the healthy wild-type controls, implying partial but significant metabolic recovery. In contrast, serum and cecum samples showed treatment-associated alterations that did not indicate metabolic normalization, while cortex and feces showed minimal changes. Given the substantial functional improvement in the hippocampus, particularly in relation to tau pathology and memory, we focused our subsequent metabolomic analyses on this brain region to better understand metabolic link between the gut and brain communication.

In the hippocampus, SRK414 led to significant restoration of organoheterocyclic compounds, most notably KYNA, which showed the largest effect size among up-regulated metabolites. Hippocampal KYNA levels strongly correlated with serum KYN and cecal TRP, suggesting a functional metabolic linkage along the gut-brain axis. Specifically, given that the SRK414-supplemented diet provides a supply of tryptophan (fig. S7, A and B), the decrease in cecal tryptophan, coupled with an increased serum KYN/TRP ratio (an indicator of KYN pathway activation, [56, 57]) may reflect increased gut TRP consumption and greater engagement of the kynurenine pathway. Additionally, the increased hippocampal KYNA/KYN ratio, together with elevated KYNA/3-OHKYN ratio, is consistent with a preferential metabolic shift toward the neuroprotective KYNA branch rather than the neurotoxic arm of kynurenine pathway. This metabolic shift toward KYNA may be driven by two plausible explanations. First, SRK414 administration significantly increased levels of 2-keto-3-deoxy-D-gluconic acid (KDG) in cecum and serum (p < 0.05; fold change: 2.9 in cecum/1.48 in serum; fig. S8, A and B). KDG is a 2-oxoacid that serves as an amino group acceptor required for KAT enzymes to produce KYNA [60]. Furthermore, the observed up-regulation of pyridoxal kinase (PDXK, a key enzyme for the KAT cofactor, fig. S9A), together with increased KDG and kynurenine levels may support a metabolic environment favorable to KYNA production. Second, the mitigation of intestinal inflammation by SRK414 may contribute to attenuated hippocampal gliosis and reduced KMO-driven neurotoxic signaling. Although these indices do not substitute for direct measurements of PLP levels or KAT/KMO activity, they support the interpretation that SRK414 reshaped the hippocampal metabolic context in a manner favorable to KYNA accumulation.

Beyond its selective enrichment, KYNA exhibits potent neuroprotective and anti-inflammatory properties. Functional validation experiments demonstrated that KYNA treatment reduced tau phosphorylation in AD patient-derived neurons and suppressed pro-inflammatory cytokine production (TNF-α, IL-1β, and IL-18) in Aβ-stimulated primary microglial cultures. These findings raise the possibility that accumulated KYNA may help sustain this neuroprotective state, in part by decreasing pro-inflammatory cytokines. Mechanistically, KYNA activates AMPK, promoting fatty acid metabolism through inhibition of acetyl-CoA carboxylase (ACC) and upregulation of carnitine palmitoyltransferase I (CPT1) activity [61]. In line with this mechanism, SRK414 increased hippocampal levels of isovaleryl-L-carnitine, isovalerate, sebacic acid, and 12-hydroxystearic acid, indicative of enhanced fatty acid metabolism. KYNA may also engage additional signaling pathways beyond AMPK. Previous studies have reported that KYNA can attenuate excitatory neurotransmission via NMDA receptor antagonism and modulate immune responses by engaging signaling pathways associated with aryl hydrocarbon receptor (AhR) and G protein-coupled receptor 35 (GPR35) [62, 63].

Importantly, KYNA treatment markedly reduced LD accumulation in AD patient-derived neurons, which was paralleled by a reduction in hippocampal LDs in vivo following SRK414 administration. LD accumulation, linked to tau pathology and impaired energy metabolism, promotes microglial dysfunction and inflammation [64]. Therefore, KYNA-mediated reduction in neuronal LDs may not only protect neurons but also mitigate chronic microglial inflammation and preserve microglial homeostasis. In addition to KYNA, SRK414 increased hippocampal levels of indole-3-acetic acid (IAA) and its precursor, indolelactic acid in the serum, consistent with systemic alterations in tryptophan-related pathways. IAA contributes to neuroprotection through anti-inflammation and antioxidant mechanisms [65–67]. These findings align with the transcriptional recovery observed in the hippocampus following SRK414 treatment, marked by enhanced neuroprotective signatures and reduced inflammatory responses.

While the hippocampus showed the most pronounced metabolic recovery, cortical tissues also showed modest level of metabolic restoration, particularly in short chain fatty acids (SCFAs) (fig. S10, A-C). SCFAs are known to support microglial function [68–70] which relies on mitochondrial OXPHOS [71, 72]. In AD, chronic Aβ exposure induces microglial metabolic exhaustion and impairs clearance function [41]. Consistent with this, SRK414 treatment restored cortical OXPHOS-related gene expression, suggesting improved microglial mitochondrial fitness and potential enhancement of Aβ clearance.

An important consideration in interpreting our transcriptomic datasets is that RNA-sequencing was performed on bulk tissues collected at a single end-point after chronic SRK414 administration. In bulk tissue RNA-sequencing, differential expression can reflect both cell-intrinsic transcriptional regulation and shifts in cell-type composition or activation states. Because our sampling captures a single terminal time point, we cannot assign temporal order or causality to individual DEGs and therefore focus on coordinated pathway-level changes supported by orthogonal phenotypic readouts.

Consistent with the central findings of this study, oral administration of *Limosilactobacillus fermentum* SRK414 increased levels of circulating KYN, hippocampal KYNA, and cortical short-chain fatty acids (SCFAs), providing a plausible mechanistic link between gut responses and CNS transcriptomic changes. Within this framework, transcriptomic alterations in the colon are interpreted as relatively proximal responses, whereas changes observed in the brain are more consistent with downstream adaptations.

In the colon, suppression of inflammatory signatures and antigen processing or presentation–related genes together with reduced Cd4 expression and decreased TNF-α protein levels is consistent with modulation of local immune activation. In contrast, restoration of neuronal, synaptic, and metabolic gene modules in the hippocampus and cortex is more likely to reflect downstream remodeling associated with improved inflammatory or metabolic conditions.

Notably, attenuation of immune-response gene programs in the hippocampus together with reduced Iba1 immunoreactivity is consistent with the possibility that part of the CNS transcriptomic changes are secondary to altered immune and microglial activation states. Future time-resolved and cell-type–resolved approaches will be required to distinguish early or primary effects from later or indirect responses.

Despite these notable findings, several limitations should be acknowledged. First, although SRK414 treatment elicited broad neuroprotective effects, the complex interactions within the gut microbial ecosystem raise the possibility that other microbial taxa or metabolites may contribute to the observed outcomes. Second, although intestinal permeability was assessed functionally, we did not evaluate canonical tight-junction markers such as ZO-1, occludin, and claudins. Therefore, the molecular basis of the barrier-related effects of SRK414 could not be directly established. Third, circulating cytokines and other inflammatory mediators were not quantified in this study. Therefore, we were unable to directly assess whether SRK414 modulates systemic inflammation. Fourth, we did not manipulate the kynurenine pathway in vivo, including supplementation with KYN/KYNA or enzyme-targeted approaches, which limits causal inference regarding pathway directionality. Fifth, although KYNA emerged as a key candidate mediator, its downstream mechanisms, including AhR and GPR35 signaling and NMDA receptor antagonism, remain to be elucidated. Finally, as our study was conducted in a mouse model, further investigation is required to evaluate the translatability of these findings to human AD pathology, considering species-specific differences in microbiota composition and host physiology.

**Fig. 7:**
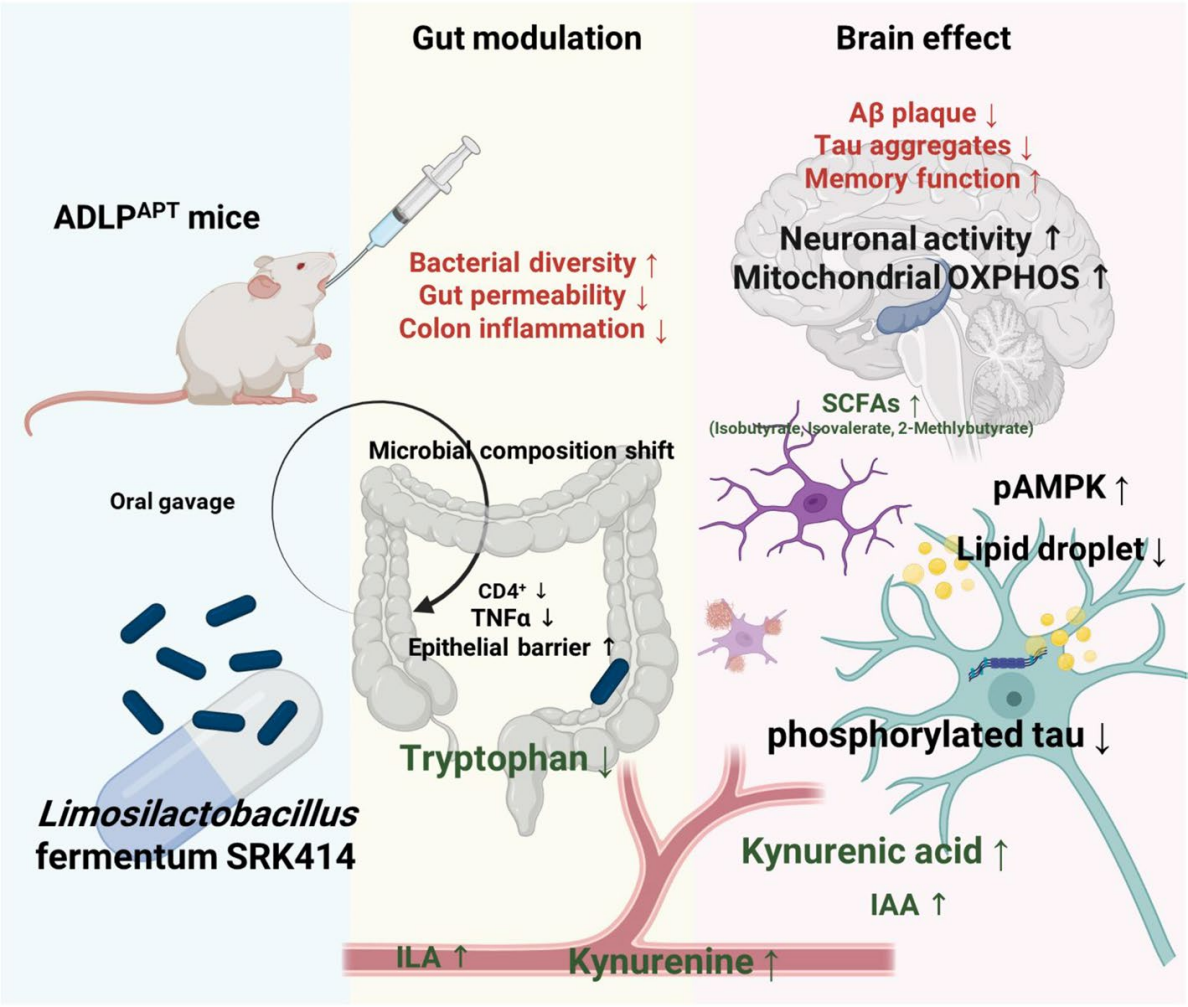
Graphic summary. Oral administration of *Limosilactobacillus fermentum* SRK414 elevates hippocampal KYNA and cortical short-chain fatty acids (SCFAs), which act along the gut–brain axis to enhance neuronal and microglial resilience in an Alzheimer’s disease (AD) mouse model

In conclusion, our findings delineate a metabolite-centered mechanism by which *Limosilactobacillus fermentum* SRK414 modulates the gut–brain axis to confer neuroprotection in Alzheimer’s disease: by reprogramming the tryptophan–kynurenine pathway, SRK414 elevates hippocampal KYNA, which activates AMPK-dependent lipid oxidation, suppresses neuroinflammation, and reduces tau phosphorylation, potentially leading to the restoration of neuronal and glial metabolic homeostasis and enhancing synaptic resilience (Fig. 7). This integrative host–microbe metabolic rewiring represents a mechanistically defined route through which microbiota-derived metabolites can modulate central energy metabolism and immune balance, offering a tractable therapeutic framework for multifactorial neurodegeneration.

## Supplementary Information

Supplementary Material 1.

Supplementary Material 2.

Supplementary Material 3.

Supplementary Material 4.

## Data Availability

The LC–MS/MS data generated during the current study are deposited in the MassIVE public repository under the accession number MSV000098470. The processed data is available in the supplementary materials. The supporting code can be found at the following GitHub page: https://github.com/SNUFML/AD-STUDY. 16S rRNA gene amplicon sequencing data of gut microbiota in ADLP mouse model following L. fermentum SRK414 administration are publicly available in the NCBI Sequence Read Archive (SRA) under accession number PRJNA1322104. Transcriptomic data from colon and brain tissues are available in the NCBI SRA under accession number PRJNA1332269. No custom code was used for 16S rRNA gene amplicon sequencing or transcriptomic analyses; only standard, publicly available bioinformatics tools, as described in the Methods section, were employed.
