# Supplementary figures and images for: Tryptophan–kynurenine metabolic reprogramming along the gut–brain axis alleviates Alzheimer’s pathology
Source: J Neuroinflammation. 2026 Apr 24;23:197. doi: 10.1186/s12974-026-03796-1 (PMC13248358; doi:10.1186/s12974-026-03796-1)

2A

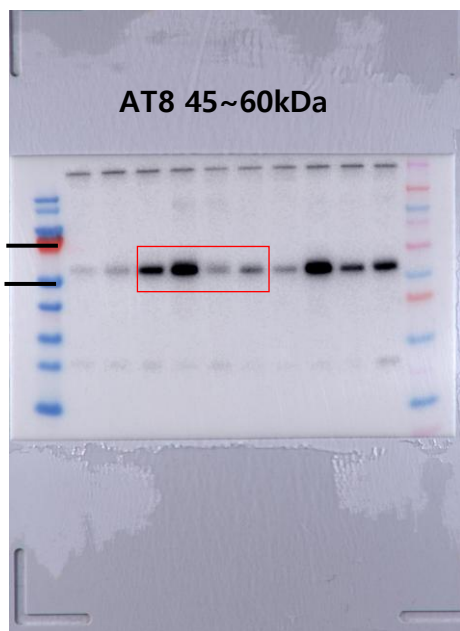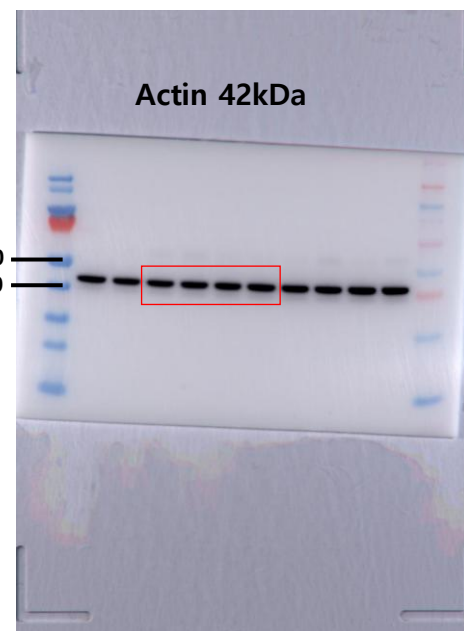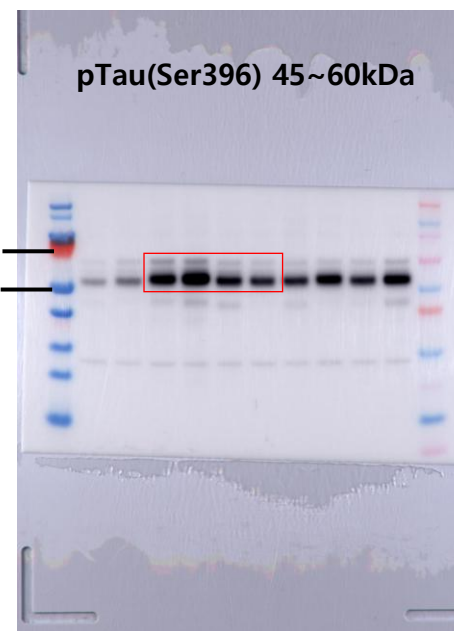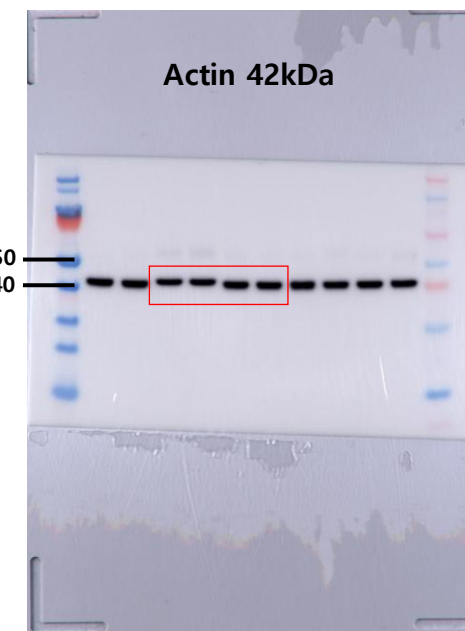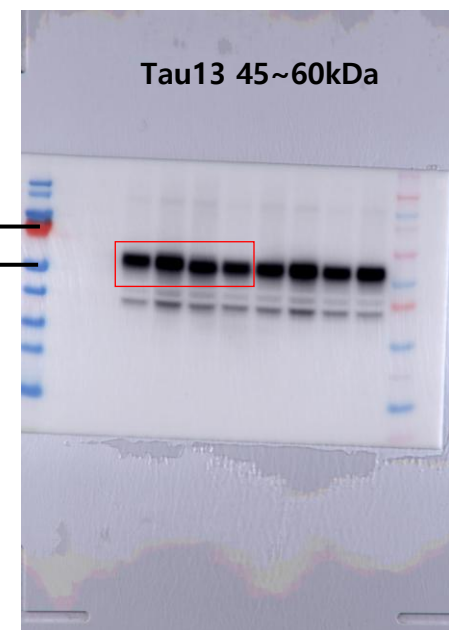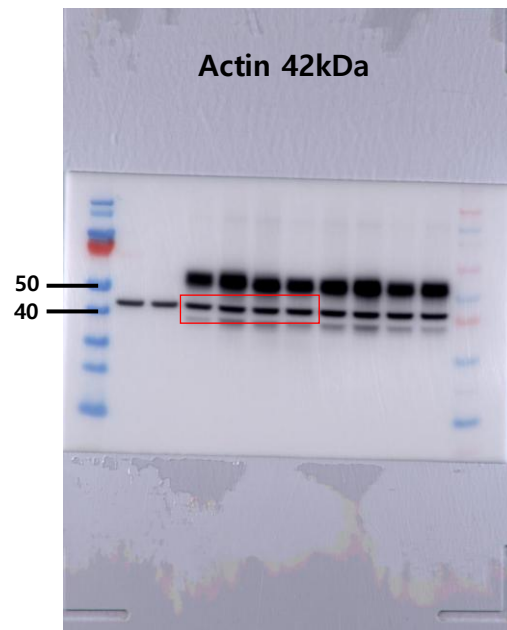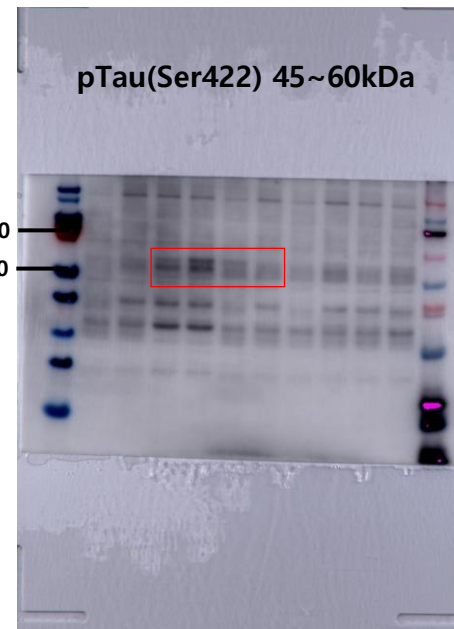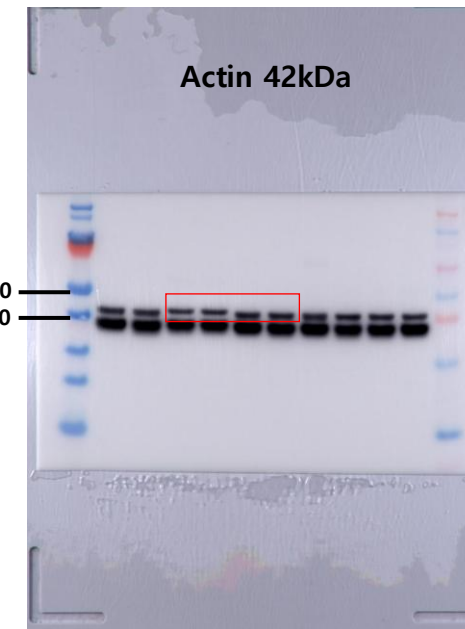

6B

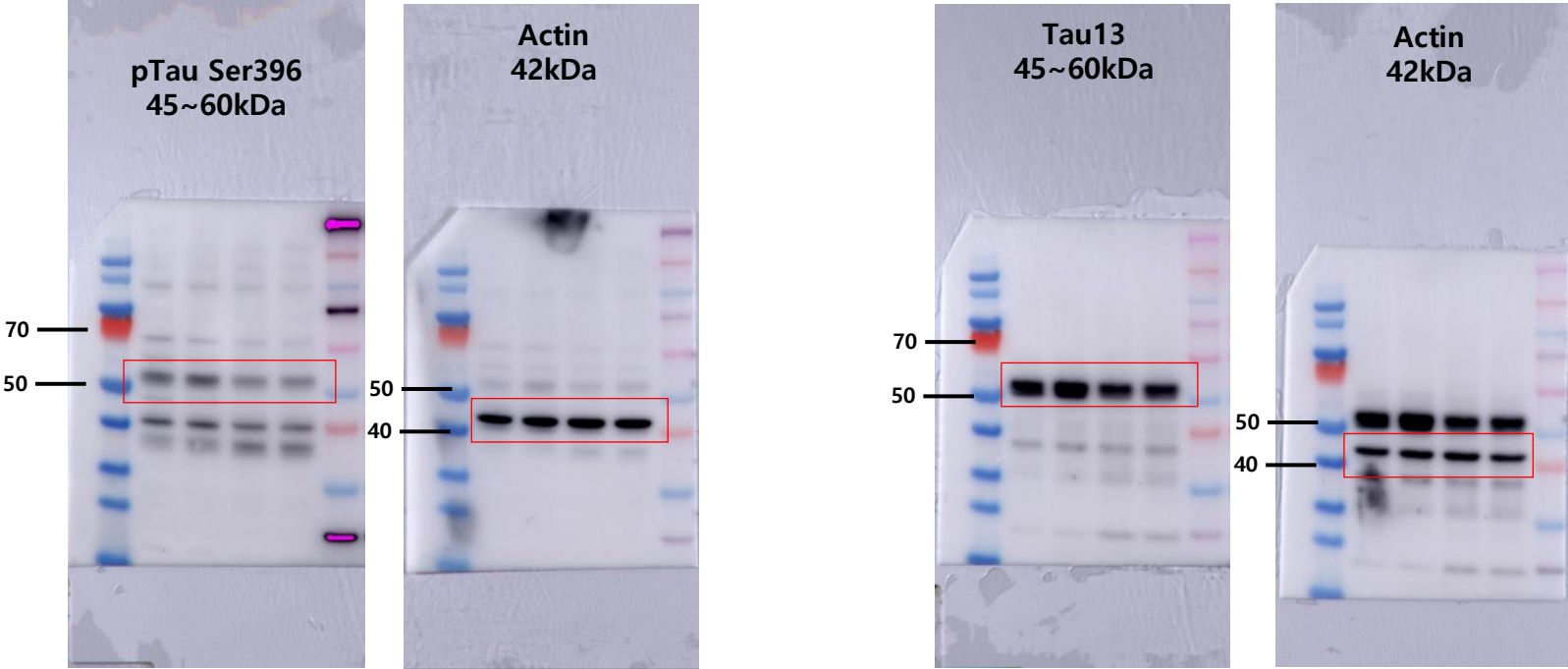

6D

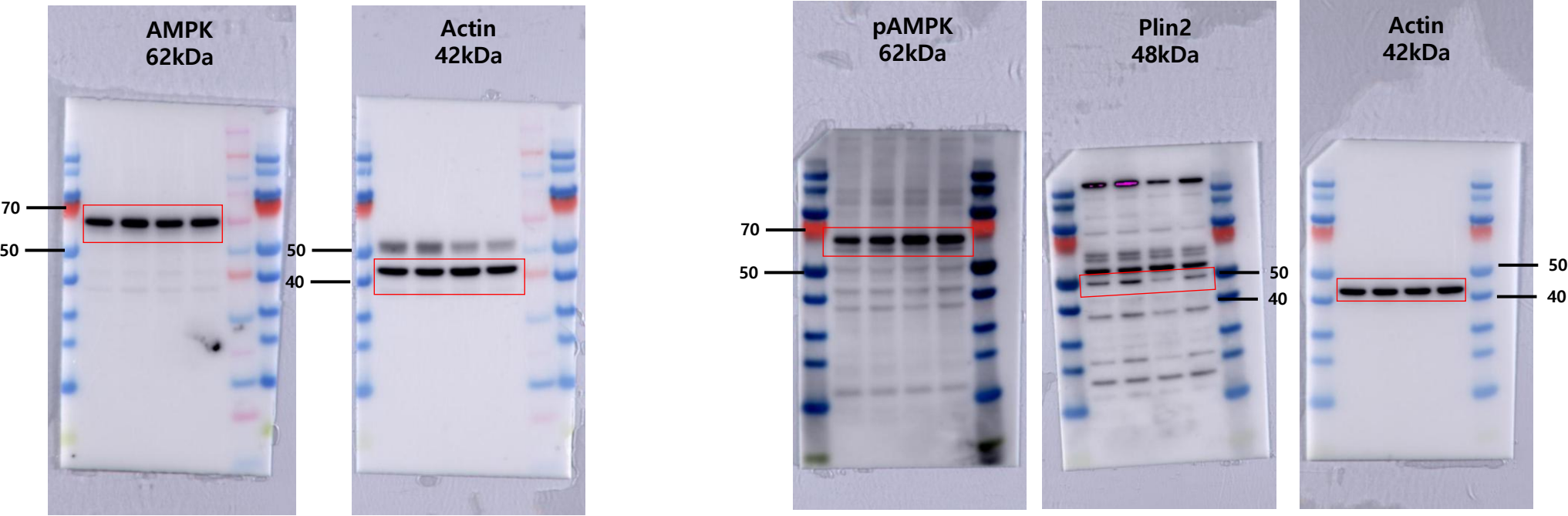

Supplement: Supplementary file 2 — Supplementary Material 2. [file 12974_2026_3796_MOESM2_ESM.pdf]
