# Supplementary material for: Tryptophan–kynurenine metabolic reprogramming along the gut–brain axis alleviates Alzheimer’s pathology
Source: J Neuroinflammation. 2026 Apr 24;23:197. doi: 10.1186/s12974-026-03796-1 (PMC13248358; doi:10.1186/s12974-026-03796-1)
Supplement: Supplementary file 3 — Supplementary Material 3. [file 12974_2026_3796_MOESM3_ESM.docx]

**Supplementary table 1. Antibody information about Immunohistochemistry**

| **Antibody** | **Catalog number, supplier** | **Dilution** |
| --- | --- | --- |
| 4G8 | SIG-39240, Covance | 1:700 |
| AT8 | MN1020, Thermofisher | 1:200 |
| Iba1 | 019-19741, Wako | 1:500 |
| Plin2 | ab52356, Abcam | 1:200 |

**Supplementary table 2. Antibody information about Immunocytochemistry**

| **Antibody** | **Catalog number, supplier** | **Dilution** |
| --- | --- | --- |
| Plin2 | ab52356, Abcam | 1:500 |
| MAP2 | Ab5392, Abcam | 1:5000 |

**Supplementary table 3. List of primers used in the study**

| Gene | Primer sequence |
| --- | --- |
| mIL-18 | F: 5’-GAC TCT TGC GTC AAC TTC AAG G-3’  R: 5’-CAG GCT GTC TTT TGT CAA CGA-3’ |
| mIFN-γ | F: 5’-GCC ACG GCA CAG TCA TTG A-3’  R: 5’-TGC TGA TGG CCT GAT TGT CTT-3’ |
| mTNF-α | F: 5’-GCC TCT TCT CAT TCC TGC TT-3’  R: 5’-TGG GAA CTT CTC ATC CCT TTG-3’ |
| m18s | F: 5’-GGA TGT GAA GGA TGG GAA GT-3’  R: 5’-CCC TCT ATG GGC TCG AAT TT-3’ |

**Supplementary table 4. Antibody information about Westernblot**

| **Antibody** | **Catalog number, supplier** | **Dilution** |
| --- | --- | --- |
| Tau13 | ab19030, Abcam | 1:2000 |
| Phospho-Tau  Ser202, Thr205 [clone AT8] | MN1020, Invitrogen | 1:2000 |
| Phospho-Tau (Ser396) Polyclonal Antibody | 44-752G,Invitrogen | 1:1000 |
| Phospho-Tau (Ser422) Polyclonal Antibody | 44-764G, Invitrogen | 1:1000 |
| Plin2 | ab52356, Abcam | 1:500 |
| β-actin | #3700, Cell Signaling Technology | 1:2000 |

**Supplementary table 5. Effect size estimates, 95% confidence intervals for group differences, and observed post hoc power for selected key in vivo efficacy endpoints**

| **Outcome measure** | **Comparison** | **Mean difference**  **(95% CI)** | **Effect size**  **Magnitude**  **(\|Hedges’ g\|)** | **Observed post hoc power** |
| --- | --- | --- | --- | --- |
| **Fig. 2F,**  Y-maze spontaneous alternation | ADLP^APT^+ SRK414 vs ADLP^APT^ | +18.22  (9.37 to 27.07) | \|Hedges’ g\| = 1.59 | 0.98 |
| **Fig. 1G**  hippocampal Aβ burden | ADLP^APT^+ SRK414 vs ADLP^APT^ | -30.34  (-51.22 to -9.46) | \|Hedges’ g\| = 1.12 | 0.82 |
| **Fig. 2D**  hippocampal AT8 immunoreactivity | ADLP^APT^+ SRK414 vs ADLP^APT^ | -64.06  (-127.60 to -0.50) | \|Hedges’ g\| = 0.80 | 0.52 |
