# Supplementary material for: Tryptophan–kynurenine metabolic reprogramming along the gut–brain axis alleviates Alzheimer’s pathology
Source: J Neuroinflammation. 2026 Apr 24;23:197. doi: 10.1186/s12974-026-03796-1 (PMC13248358; doi:10.1186/s12974-026-03796-1)
Supplement: Supplementary file 4 — Supplementary Material 4. [file 12974_2026_3796_MOESM4_ESM.docx]

**Supplementary Information for**

**Tryptophan–Kynurenine Metabolic Reprogramming Along the Gut–Brain Axis Alleviates Alzheimer’s Pathology**

Hyunjung Choi^1†^, Seok Beom Hong^1,2†^, Yumi Kim^3†^, Hyunchae Joung^4^, Yukyung Choi^4^, Jiah Cha^4^,Ji Yong Park^5,6,7,8^, Yun-Sang Lee^5,6,7,8,9^, Hayoung Choi^1,2^, Jong Won Han^1,2^, Kyung Hwan Kim^4^, Chang Hun Shin^4^**,** Do Yup Lee^3,10*^, Inhee Mook-Jung^1,2*^

**Affiliation**

^1^ Convergence Dementia Research Center, Medical Research Center, Seoul National University, Seoul, 03080, Republic of Korea

^2^ Department of Biomedical Science, College of Medicine, Seoul National University, Seoul, 03080, Republic of Korea

^3^Department of Agricultural Biotechnology, Seoul National University, Seoul, Republic of Korea

^4^Central Research Institute, Chong Kun Dang Bio , Ansan 15604, Republic of Korea

^5^Department of Nuclear Medicine, Seoul National University Hospital, 03080, Seoul, Republic of Korea

^6^Cancer Research Institute, Seoul National University, 03080, Seoul, Republic of Korea

^7^Department of Nuclear Medicine, College of Medicine, Seoul National University, 03080, Seoul, Republic of Korea

^8^Institute of Radiation Medicine, Medical Research Center, College of Medicine, Seoul National University, 03080, Seoul, Republic of Korea

^9^Department of Molecular Medicine and Biopharmaceutical Sciences, Graduate School of Convergence Science and Technology, Seoul National University, Seoul 08826, Republic of Korea

^10^Center for Food and Bioconvergence, Research Institute for Agricultural and Life Sciences, Interdisciplinary Programs in Agricultural Genomics, Seoul National University, Seoul, 08826, Republic of Korea

^†^ These authors contributed equally to this work

^*^Correspondence to:

Inhee Mook-Jung Ph.D., Seoul National University College of Medicine, 103 Daehak-ro, Jongno-gu, Seoul, 03080, Korea, inhee@snu.ac.kr

Do Yup Lee Ph.D., Department of Agricultural Biotechnology, Seoul National University, Seoul, Republic of Korea, rome73@snu.ac.kr

**Contents**

Supplementary figures and figure legends

**Supplementary figures and Figure legends**

**fig S1. *L. fermentum* SRK414 does not affect body weight or general locomotor activity in ADLP^APT^ mice**

**(A)** Measurements of body weight at the time of behavioral testing, following the completion of SRK414 administration.
**(B)** Locomotor activity assessed by the total distance traveled in the open field test.

**(C)** Percentage of novel object preference assessed by the novel object recognition test. All data are presented as mean ± SEM with each data point representing a mouse. Statistical analysis was determined using one-way ANOVA followed by Tukey’s multiple comparisons test.

Sample size (biological replicates): (A), n=7 per ADLP ^WT^ mouse, n=8 per ADLP^APT^ mouse, n=7 per ADLP ^APT^+ SRK414 mouse; (B), n=7 per ADLP ^WT^ mouse, n=7 per ADLP^APT^ mouse, n=8 per ADLP^APT^+ SRK414 mouse; (C), n=25 per ADLP ^WT^ mouse, n=13 per ADLP^APT^ mouse, n=12 per ADLP ^APT^+ SRK414 mouse. Independent experimental repeats: (A, N = 1; B, N=1; C, N= 2).

**fig S2.** ***L. fermentum* SRK414-induced cortical and hippocampal transcriptional remodeling towards neuroprotection in ADLP^APT^ mice**

**(A)** Venn diagram of Differentially Expressed Genes (DEGs) in the hippocampus. **(B–E)** Relative expression and protein–protein interaction (PPI) network analysis of SRK414-responsive genes in the cortex (B and D) and hippocampus (C and E). Violin plots represent data distribution with individual data points shown. Statistical analysis was performed using one-way analysis of variance (ANOVA) followed by Dunnett’s multiple comparisons test. p < 0.05 was considered statistically significant.
**(F)** Representative images of Iba1 in the hippocampus of ADLP^APT^ mice or ADLP^APT^+SRK414 mice. Scale bar, 200 μm.
**(G)** Quantification of Iba1 signals. Data are presented as mean ± SEM with each data point representing a mouse. Statistical significance was determined using a two-tailed unpaired t test.

Sample size (biological replicates): (A-E), n=8 per ADLP^WT^ mouse, n=5 per ADLP^APT^ mouse, n=6 per ADLP^APT^+ SRK414 mouse; (F-G), n=15 per ADLP^APT^ mouse , n=12 per ADLP^APT^ + SRK414 mouse). Independent experimental repeats: (A-E, N = 1; F-G, N=2).

**fig S3.** ***L. fermentum* SRK414 restores mitochondrial gene in the cortex of ADLP^APT^ mice**

**(A)** Venn diagram of Differentially Expressed Genes (DEGs) in the cortex.
**(B)** Gene set enrichment analysis (GSEA) plot of cortex showing enrichment of oxidative phosphorylation pathway.
**(C–F)** Relative expression levels of mitochondrial genes (NADH dehydrogenase, Succinate dehydrogenase, Cytochrome c oxidase/reductase, ATP synthase) in the cortex compared to ADLP^WT^. Data are expressed as mean ± standard deviation (SD), with individual values overlaid. Statistical significance was determined using two-way analysis of variance (ANOVA) followed by Tukey’s post hoc test for multiple comparisons (C, E, and F). Statistical analysis was performed using an unpaired two-tailed t-test. (D) p < 0.05 was considered statistically significant.

Sample size (biological replicates): (C-F), n=5 per ADLP^APT^ mouse , n=6 per ADLP^APT^ + SRK414 mouse. Independent experimental repeats: N = 1.

**fig S4.** **Barrier-related analyses of** ***L. fermentum* SRK414 in ADLP^APT^ mice and cytokine-challenged apical-out colon organoids**

**(A)** Relative serum FITC–dextran fluorescence measured 2 hr after oral gavage in ADLP^APT^ and SRK414-treated ADLP^APT^ mice. Data are presented as mean ± SEM. Statistical significance was determined using a two-tailed unpaired t test.

**(B)** Quantification of FITC–dextran intensity in apical-out colon organoids. Organoids were pre-exposed to TNF-α and IFN-γ for 48 hr to induce barrier dysfunction, followed by 24 hr incubation with live or heat-killed SRK414. Each dot represents an individual organoid, and bars indicate mean ± SEM. Statistical significance was determined using one-way ANOVA followed by Tukey’s multiple comparisons test.

**(C)** Representative bright-field and corresponding FITC–dextran fluorescence images of apical-out colon organoids under vehicle (Veh), TNF-α/IFN-γ (T+I), T+I plus live SRK414, and T+I plus heat-killed SRK414 conditions. Scale bar, 50 μm

Sample size (biological replicates): (A), n=9 per ADLP^APT^ mouse , n=6 per ADLP^APT^ + SRK414 mouse; (B-C) n=47 organoid per condition. Independent experimental repeats: (A, N = 1; B-C, N=2)

**fig S5.** ***L. fermentum* SRK414 modulates metabolite profiles across multiple biological compartments in ADLP^APT^ mice**

**(A–D)** PCA score plots and box plots of PC1 and PC2 in the cortex (A), serum (B), cecum (C), and feces (D). Asterisks indicate statistical significance (* *P* < 0.05; ** *P* < 0.01; Mann–Whitney U test).
**(E–F)** SRK414-modulated indole derivatives: (E) indole-3-acetic acid (hippocampus), (F) indolelactic acid (serum). Asterisks indicate statistical significance (* *P* < 0.05, ** *P* < 0.01; Mann–Whitney U test). **(G)** Spearman correlation analysis of significantly altered metabolites across the hippocampus, serum, and cecum. Metabolites were selected based on *P* < 0.05, |fold change| ≥ 1.5, and |Hedge’s g| ≥ 0.8. Edges indicate significant correlations (*P* < 0.05); red lines represent positive and blue lines negative correlations. Inner circle color indicates direction of change with SRK414 (orange: increased; blue: decreased). **(H)** Serum KYN/TRP ratio. **(I)** 3-hydroxykynurenine (3-OHKYN) (hippocampus). **(J)** Correlation scatter plot of hippocampal kynurenic acid (KYNA) and 3-hydroxykynurenine (3-OHKYN) in response to SRK414 treatment. **(K-M)** Ratios of kynurenine pathway metabolites in the hippocampus: (K) 3-OHKYN/KYN, (L) KYNA/KYN, (M) KYNA/3-OHKYN

Sample size (biological replicates): (A-M), n=8 per ADLP^WT^ mouse, n=5 per ADLP^APT^ mouse, n=6 per ADLP^APT^+ SRK414 mouse. Independent experimental repeats: N = 1

**fig S6. KYNA suppresses microglial inflammatory cytokine production and *L. fermentum* SRK414 reduces lipid droplet accumulation in the hippocampus of ADLP^APT^ mice**

**(A)** Schematic representation of Aβ and KYNA treatment in primary microglia. Primary microglia were treated for 24 h with vehicle (Veh), Aβ (4μM), Aβ (4μM) plus KYNA (50μM), or KYNA (50μM) alone. Figure generated using Biorender.
**(B–D)** Relative mRNA expression levels of *TNF-α* (B), *IL-1β* (C), and *IL-18* (D) in primary microglia under indicated treatment condition (Veh, Aβ 4μM, Aβ 4μM + KYNA 50μM, KYNA 50μM; 24 h), normalized to control. Data are presented as mean ± SEM. Statistical analysis was determined using one-way ANOVA followed by Tukey’s multiple comparisons test. **(E)** Representative images of Plin2 in the hippocampus of ADLP^WT^ mice, ADLP^APT^ mice or ADLP^APT^+SRK414 mice. Scale bar, 200 μm.
**(F)** Quantification of Plin2 intensity and area fraction. Data are presented as mean ± SEM with each data point representing a mouse. Statistical analysis was determined using one-way ANOVA followed by Tukey’s multiple comparisons test.

Sample size (biological replicates): (A-D) n=3 per group; (E-F) n=5 per ADLP^WT^ mouse, n=5 per ADLP^APT^ mouse, n=5 per ADLP^APT^+ SRK414 mouse. Independent experimental repeats: N = 1.

**fig S7. The Probiotic powder** (***L. fermentum* SRK414), used as the feeding material for in vivo studies, contains tryptophan**

(A) Extracted ion chromatogram (XIC) of tryptophan obtained in positive ion mode (M+H+) from the SRK414-supplemented diet. (B) MS/MS spectra, blue traces correspond to the sample, while red traces correspond to the library compound

**fig S8. *L. fermentum* SRK414 modulates 2-keto-3-deoxy-D-gluconic acid in cecum and serum**

(A) 2-keto-3-deoxy-D-gluconic acid in cecum, (B) 2-keto-3-deoxy-D-gluconic acid in serum. Asterisks indicate statistical significance (* *P* < 0.05, ** *P* < 0.01; Mann–Whitney U test).

Sample size (biological replicates): (A-B), n=8 per ADLP^WT^ mouse, n=5 per ADLP^APT^ mouse, n=6 per ADLP^APT^+ SRK414 mouse. Independent experimental repeats: N = 1

**fig S9. Hippocampal expression of kynurenine pathway–related genes in ADLP mice**

**(A)** Hippocampal PDXK RNA-seq expression of kynurenine pathway–related genes, shown as log2(FPKM): Individual dots represent biologically independent mice, and bars indicate mean ± SEM. Statistical significance was determined by one-way ANOVA followed by Tukey’s multiple-comparisons test, unless otherwise indicated. Asterisks denote significance identified by Tukey’s multiple-comparisons test (**P < 0.01), and # denotes P < 0.05 by two-tailed unpaired Student’s t-test.

Sample size (biological replicates): (A), n=8 per ADLP^WT^ mouse, n=5 per ADLP^APT^ mouse, n=6 per ADLP^APT^+ SRK414 mouse. Independent experimental repeats: N = 1

**fig S10. *L. fermentum* SRK414 modulates cortical short-chain fatty acids in ADLP^APT^ mice**

**(A-C)** SRK414-induced changes in cortical short-chain fatty acids: (A) isobutyrate, (B) isovalerate, (C) 2-methylbutyrate. Asterisks indicate statistical significance (* *P* < 0.05, ** *P* < 0.01; Mann–Whitney U test).

Sample size (biological replicates): (A-C), n=8 per ADLP^WT^ mouse, n=5 per ADLP^APT^ mouse, n=6 per ADLP^APT^+ SRK414 mouse. Independent experimental repeats: N = 1

**
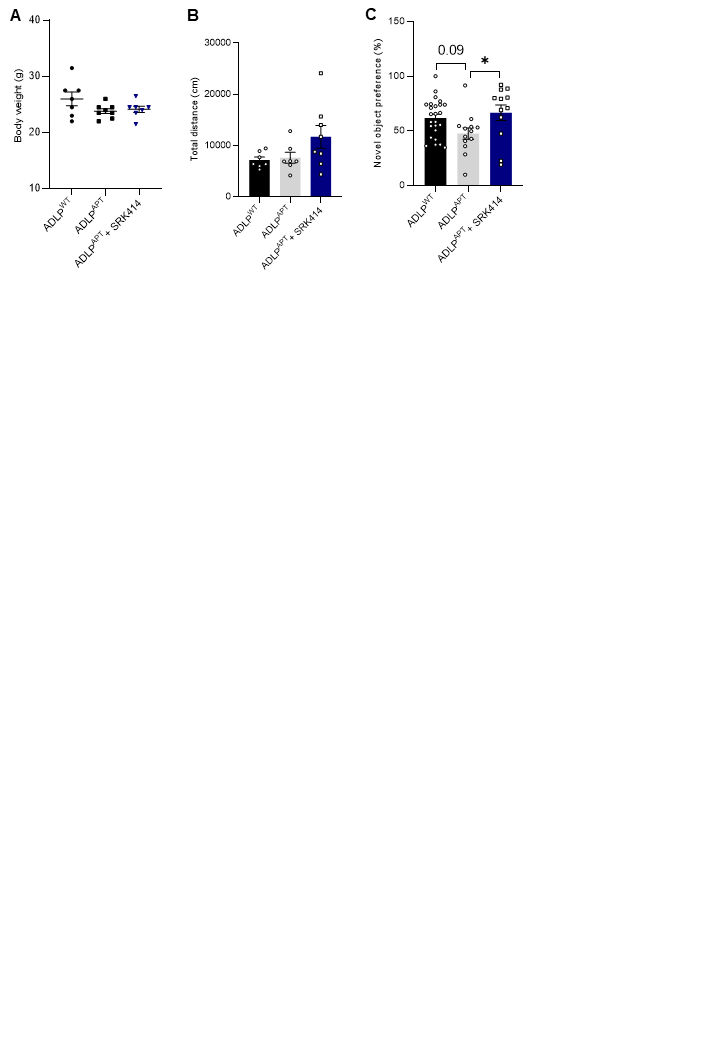
fig S1. *L. fermentum* SRK414 does not affect body weight or general locomotor activity in ADLP^APT^ mice**

**
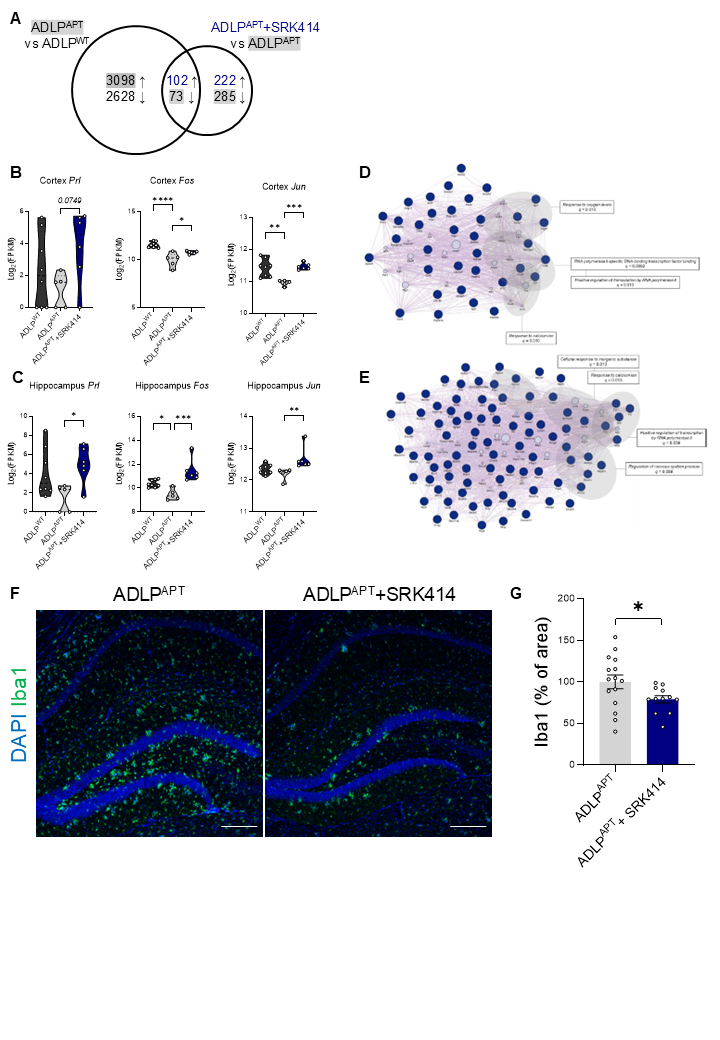
**

**fig S2.** ***L. fermentum* SRK414-induced cortical and hippocampal transcriptional remodeling towards neuroprotection in ADLP^APT^ mice**

**
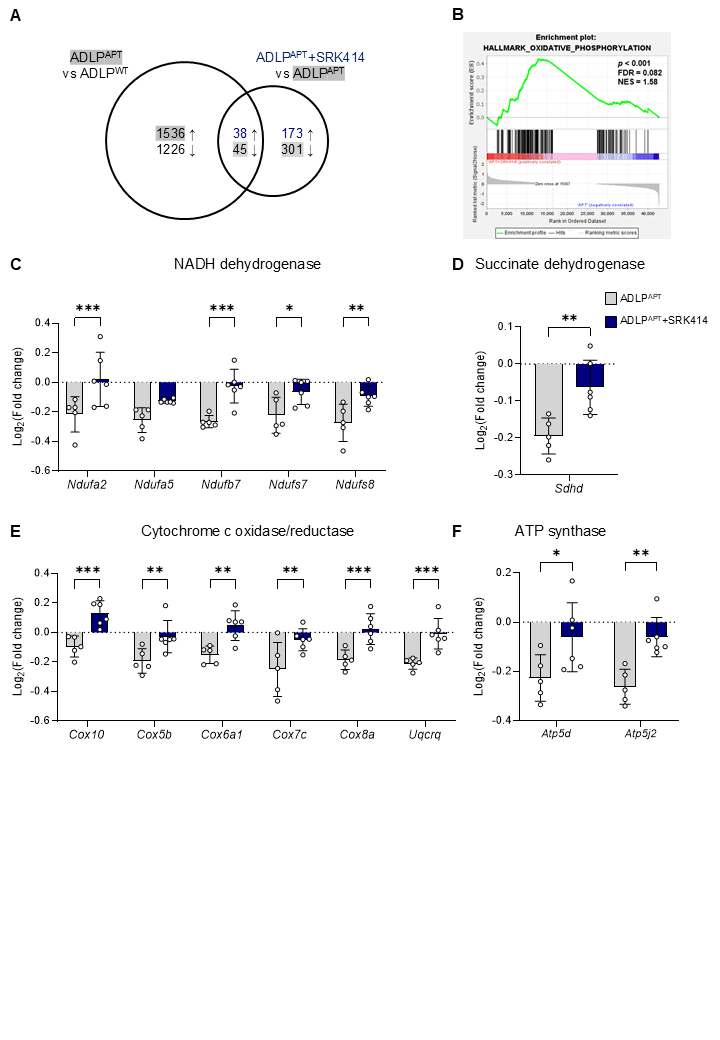
**

**fig S3.** ***L. fermentum* SRK414 restores mitochondrial gene in the cortex of ADLP^APT^ mice**

**
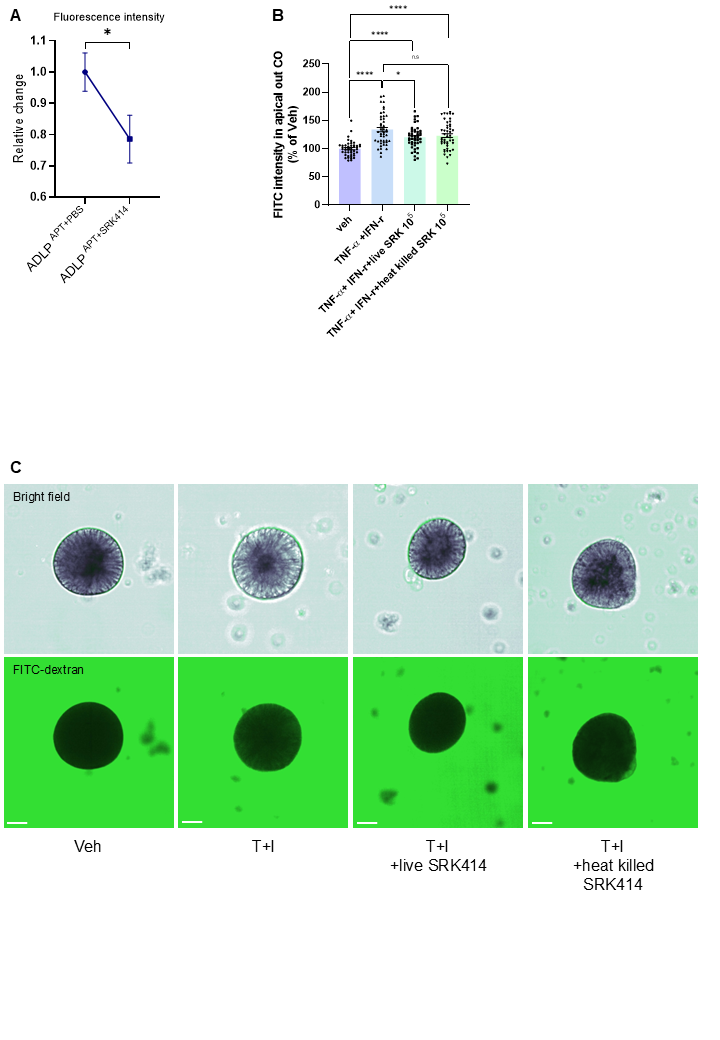
**

**fig S4.** **Barrier-related analyses of** ***L. fermentum* SRK414 in ADLP^APT^ mice and cytokine-challenged apical-out colon organoids**

**
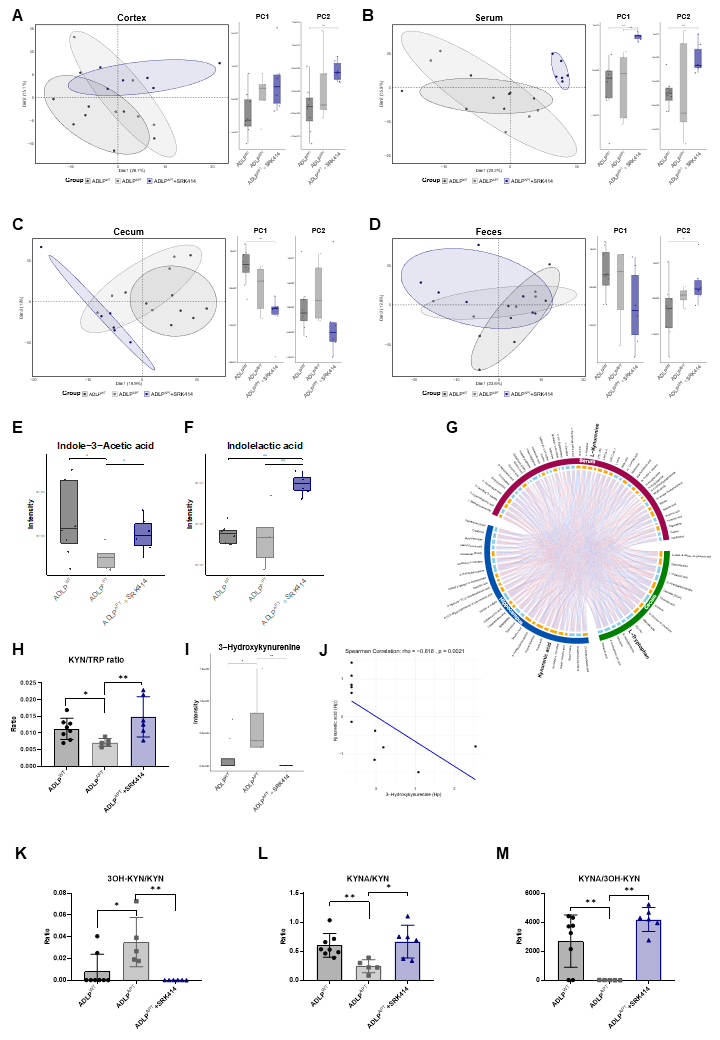
**

**fig S5.** ***L. fermentum* SRK414 modulates metabolite profiles across multiple biological compartments in ADLP^APT^ mice**

**
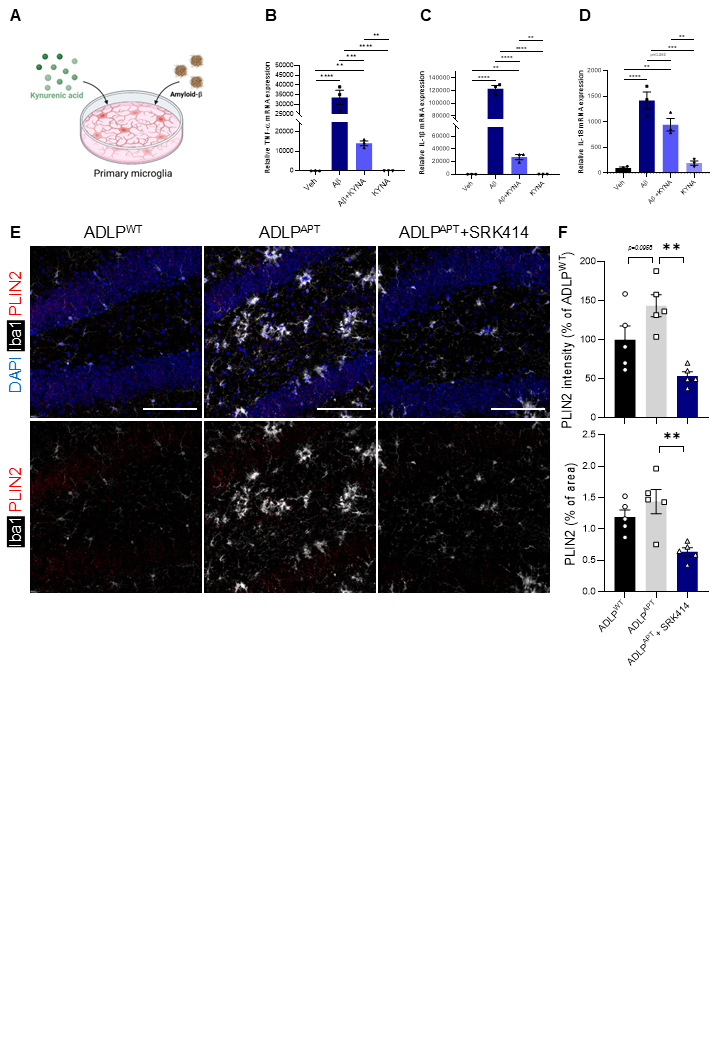
**

**fig S6. KYNA suppresses microglial inflammatory cytokine production and *L. fermentum* SRK414 reduces lipid droplet accumulation in the hippocampus of ADLP^APT^ mice**

**
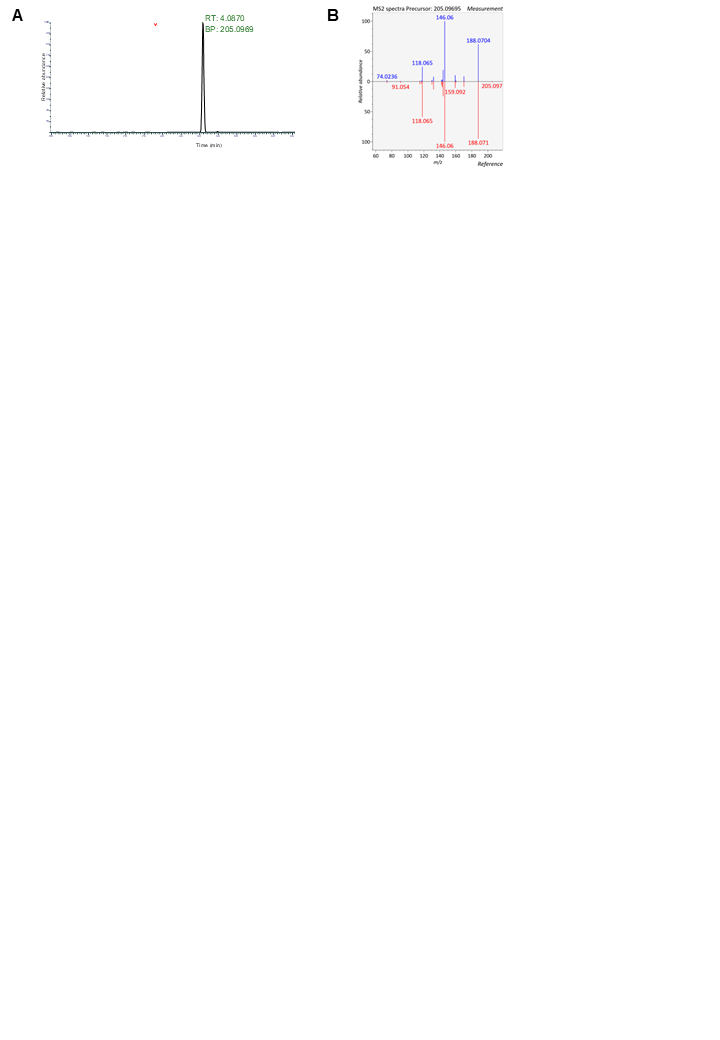
fig S7. The Probiotic powder** (***L. fermentum* SRK414), used as the feeding material for in vivo studies, contains tryptophan**

**
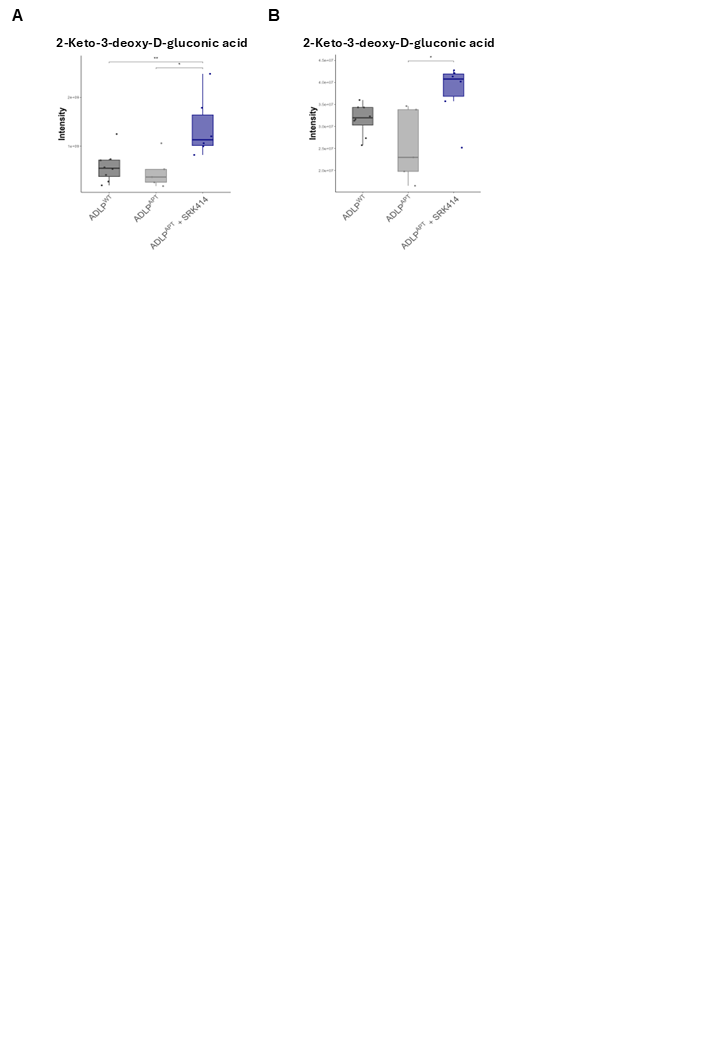
fig S8. *L. fermentum* SRK414 modulates 2-keto-3-deoxy-D-gluconic acid in cecum and serum**

**
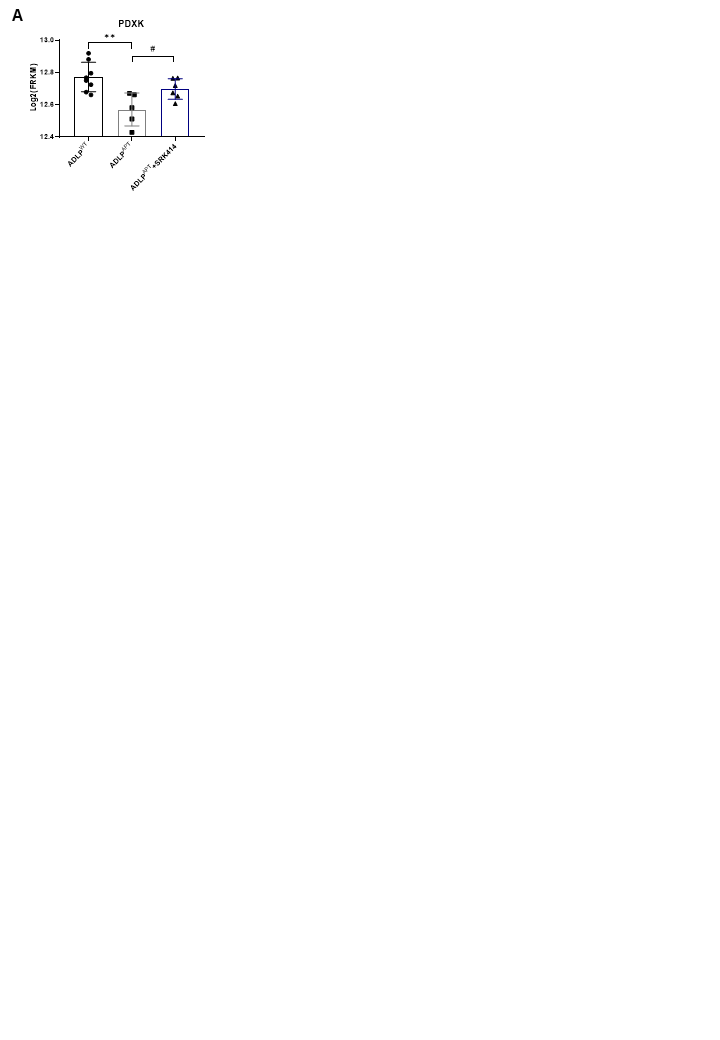
fig S9. Hippocampal expression of kynurenine pathway–related genes in ADLP mice**

**
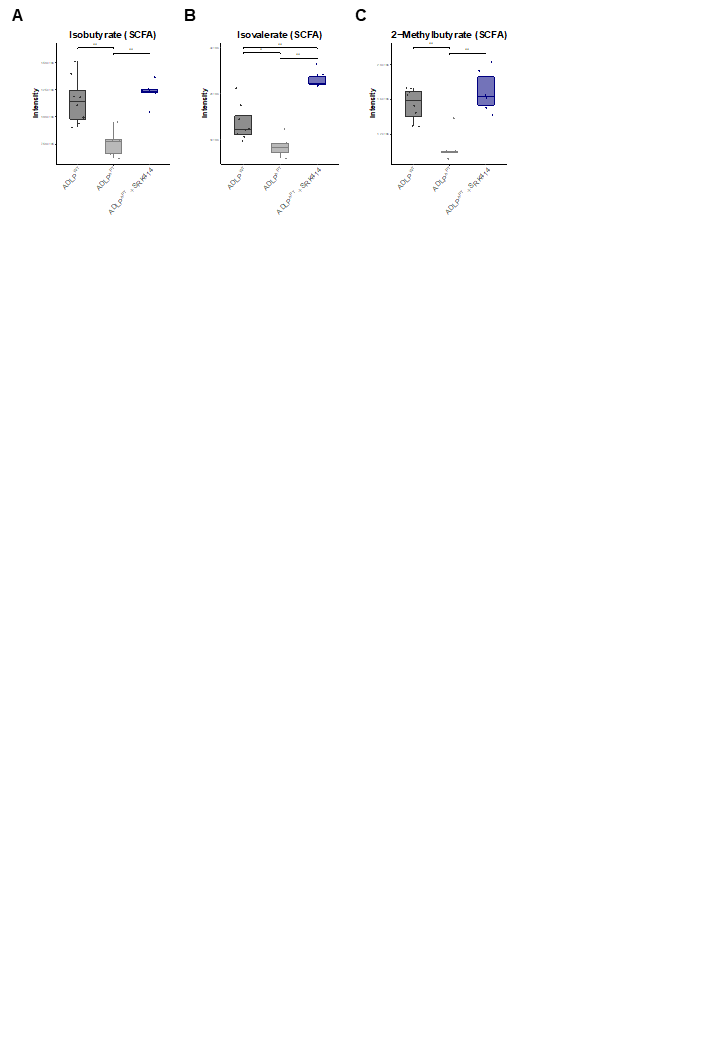
 fig S10. *L. fermentum* SRK414 modulates cortical short-chain fatty acids in ADLP^APT^ mice**
